# Supplementary material for: Multi‐Omics Factor Analysis—a framework for unsupervised integration of multi‐omics data sets
Source: Mol Syst Biol. 2018 Jun 20;14(6):e8124. doi: 10.15252/msb.20178124 (PMC6010767; doi:10.15252/msb.20178124)
Supplement: Supplementary file 1 — Appendix [file MSB-14-e8124-s001.pdf]

Appendix for

Multi-Omics factor analysis - a framework for unsupervised integration of multi-omic data sets

Ricard Argelaguet, Britta Velten, Damien Arnol, Sascha Dietrich, Thorsten Zenz, John C. Marioni, Florian Buettner, Wolfgang Huber, Oliver Stegle

Contents

1 Introduction **2**

2 Multi-Omics Factor Analysis model **2**

3 Model Inference **4**

3.1 Introduction to variational Bayes inference . . . 4

3.2 Update equations for Gaussian data . . . 5

4 Modelling and inference with non-Gaussian data **8**

5 Implementation and practical considerations for training **10**

5.1 Monitoring convergence . . . 10

5.2 Handling of missing values . . . 10

5.3 Data pre-processing . . . 10

5.4 Consistency across random initilizations . . . 10

5.5 Determining the number of factors . . . 10

5.6 Rotational invariance . . . 11

6 Supplementary Figures **13**

7 Supplementary Tables **37**

1 Introduction

Multi-Omics Factor Analysis (MOFA) is a statistical model aimed at disentangling sources of variation in multi-omics data. Here, we introduce the statistical model (section 2) and its inference procedure in more detail, both in case of Gaussian data (section 3) and non-Gaussian data (section 4). In addition, we provide practical considerations for training (section 5).

Mathematical notation

- Matrices are denoted with bold capital letters: $\mathbf{W}$
- Vectors are denoted with bold non-capital letters. If the vector comes from a matrix, two indices separated by a comma will always be shown at the bottom: the first one corresponding to the row and the second one to the column. The symbol ':' denotes the entire row/column. For instance, $\mathbf{w}_{j,:}$ refers to the entire jth row from $\mathbf{W}$ matrix.
- Scalars are denoted with non-bold non-capital letters. If the value comes from a matrix, two indices separated by a comma will always be shown at the bottom: the first one corresponding to the row and the second one to the column. For instance, $w_{j,k}$ refers to the value coming from the jth row and the kth column from the $\mathbf{W}$ matrix.
- $\mathbf{0}_k$ is a zero vector of length K.
- $\mathbf{I}_k$ is the identity matrix with rank K.
- $\mathbb{E}_q[x]$ denotes the expectation of x under the distribution q. Sometimes, when the expectations are taken with respect to the same distribution many times, to avoid cluttered notation we will use $\langle x \rangle$.
- $\mathcal{N}(x \mid \mu, \sigma)$: x follows a univariate normal distribution with mean μ and variance σ.
- $\mathcal{G}(x \mid a, b)$: x follows a gamma distribution with parameters a and b.
- $diag(\mathbf{x})$ is the diagonal operator that takes as input a vector and outputs a diagonal matrix with $\mathbf{x}$ in the diagonal.

2 Multi-Omics Factor Analysis model

Factor analysis models, also called latent variable models, are a probabilistic modelling approach which aim to reduce the dimensionality of a (big) dataset into a small set of variables which are easier to interpret and visualise. More formally, given a dataset $\mathbf{Y}$of N samples and D features, latent variable models attempt to explain dependencies between the features by means of a potentially smaller set of K unobserved (latent) factors. MOFA is a generalisation of traditional Factor Analysis where the input data consists of M matrices $\mathbf{Y}^m = [y_{nd}^m] \in \mathbb{R}^{N \times D_m}$ where each matrix m is called a view. Each view consists of non-overlapping features which usually, but not necessarily, represent different assays. The input data is then factorised as:

$$\mathbf{Y}^m = \mathbf{Z}\mathbf{W}^{mT} + \boldsymbol{\epsilon}^m, \tag{1}$$

where $\mathbf{Z} = [z_{nk}] \in \mathbb{R}^{N \times K}$ is a single matrix that contains the low-dimensional latent variables, $\mathbf{W}^m = [w_{dk}^m] \in \mathbb{R}^{D_m \times K}$ are loading matrices that relate the high-dimensional space to the low dimensional representation, and $\boldsymbol{\epsilon}^m = [\epsilon_d^m] \in \mathbb{R}^{D_m}$ denotes residual noise. We start by assuming Gaussian residuals $\boldsymbol{\epsilon}^m$, similar to standard (group) factor analysis models, while allowing for heteroscedasticity across features:

$$p(\epsilon_d^m) = \mathcal{N}(\epsilon_d^m \mid 0, 1/\tau_d^m). \tag{2}$$

This results in the following normal likelihood (for extensions to non-Gaussian settings see section 4):

$$p(y_{nd}^m) = \mathcal{N}\left(y_{nd}^m \mid \mathbf{z}_{n,:}\mathbf{w}_{d,:}^{mT}, 1/\tau_d^m\right), \tag{3}$$

where $\mathbf{w}_{d,:}^m$ denotes the d-th row of the loading matrix $\mathbf{W}^m$ and $\mathbf{z}_{n,:}$ the n-th row of the latent factor matrix $\mathbf{Z}$. For a fully probabilistic treatment we place prior distributions on the weights $\mathbf{W}^m$, the latent variables $\mathbf{Z}$ as well as on the precision of the noise $\boldsymbol{\tau}^m$. We use a standard Gaussian prior on the latent variables and a conjugate Gamma prior for the precision:

$$p(z_{n,k}) = \mathcal{N}(z_{n,k} \mid 0, 1), \tag{4}$$

$$p(\tau_d^m) = \mathcal{G}(\tau_d^m \mid a_0^\tau, b_0^\tau), \tag{5}$$

with $a_0^\tau, b_0^\tau = 1e^{-14}$ to obtain uninformative priors.

A key determinant of the model is the regularization used on the weights $\mathbf{W}^m$. MOFA encodes two levels of sparsity: a view- and factor-wise sparsity and a feature-wise sparsity. The aim of the factor- and view-wise sparsity is to identify which factors are active in which view, such that the weight vector $\mathbf{w}_{:,k}^m$ is shrunk to zero if the factor k does not drive any variation in view m. This is the general property that allows the model to disentangle the sources of variability between different assays.
In addition, we place a second layer of feature-wise sparsity whichs puts zero weights on individual features from active factors. This relies on the assumption that biological sources of variability are typically sparse, i.e. only a small number of features are "active", i. e., have non-zero weight. We achive both levels of sparsity by placing appropriate priors on the weight matrices.

Specifically, we combine an Automatic Relevance Determination (ARD) prior [9] for the view- and factor-wise sparsity with a spike-and-slab prior [10] for the feature-wise sparsity, similar to [7]. However, the spike-and-slab prior

$$p(w) = (1-\theta)\mathbb{1}_0(w) + \theta\mathcal{N}(w \mid 0, 1/\alpha) \tag{6}$$

contains a Dirac delta function, which makes the inference troublesome, here we use a re-parametrization of the weights w as a product of a Gaussian random variable $\hat{w}$ and a Bernoulli random variable s, [12, 4] resulting in the following prior:

$$p(\hat{w}_{d,k}^m, s_{d,k}^m) = \mathcal{N}(\hat{w}_{d,k}^m \mid 0, 1/\alpha_k^m)\,\mathrm{Ber}(s_{d,k}^m \mid \theta_k^m) \tag{7}$$

In this formulation α_k^m controls the strength of factor k in view m and θ_k^m controls the degree of contribution from the spike term, determining the overall feature-wise sparsity levels of factor k in view m. In order to automatically learn these parameters we use the following conjugate priors

$$p(\theta_k^m) = \mathrm{Beta}\left(\theta_k^m \mid a_0^\theta, b_0^\theta\right) \tag{8}$$

$$p(\alpha_k^m) = \mathcal{G}\left(\alpha_k^m \mid a_0^\alpha, b_0^\alpha\right), \tag{9}$$

with hyper-parameters $a_0^\theta, b_0^\theta = 1$ and $a_0^\alpha, b_0^\alpha = 1e^{-14}$ to get uninformative priors. A value of θ_k^m close to 0 implies that most of the weights of factor k in view m are shrinked to 0, which is the definition of a sparse factor. In contrast, a value of θ_k^m close to 1 implies that most of the weights are non-zero, which is the definition of a non-sparse factor.

In practice, the ARD prior yields a matrix $\boldsymbol{\alpha} \in \mathbb{R}^{M \times K}$ that defines four different types of factors:

- Factors that do not explain variation in any data set (inactive factors): all values in the corresponding columns of $\boldsymbol{\alpha}$ are large. These factors are actively removed from the model during training.
- Factors that explain variation in all data sets (fully shared factors): all M values in the corresponding columns of $\boldsymbol{\alpha}$ are small.
- Factors that explain variation in a single data set (unique factors): all values in the corresponding columns of $\boldsymbol{\alpha}$ are very large, except one.
- Factors that explain variation in a subset of data sets (partially shared factors): some values in the corresponding columns of $\boldsymbol{\alpha}$ are very large whereas others are small.

Using these prior distributions, the joint probability density function is given by

$$
\begin{aligned}
p(\mathbf{Y}, \hat{\mathbf{W}}, \mathbf{S}, \mathbf{Z}, \mathbf{\Theta}, \boldsymbol{\alpha}, \boldsymbol{\tau}) = & \prod_{m=1}^{M} \prod_{n=1}^{N} \prod_{d=1}^{D_m} \mathcal{N}\left(y_{nd}^m \,|\, \sum_{k=1}^{K} s_{dk}^m \hat{w}_{dk}^m z_{nk}, 1/\tau_d \right) \\
& \prod_{m=1}^{M} \prod_{d=1}^{D_m} \prod_{k=1}^{K} \mathcal{N}\left(\hat{w}_{dk}^m \,|\, 0, 1/\alpha_k^m\right) \text{Ber}(s_{d,k}^m | \theta_k^m) \\
& \prod_{n=1}^{N} \prod_{k=1}^{K} \mathcal{N}\left(z_{nk} \,|\, 0, 1\right) \\
& \prod_{m=1}^{M} \prod_{k=1}^{K} \text{Beta}\left(\theta_k^m \,|\, a_0^\theta, b_0^\theta\right) \\
& \prod_{m=1}^{M} \prod_{k=1}^{K} \mathcal{G}\left(\alpha_k^m \,|\, a_0^\alpha, b_0^\alpha\right) \\
& \prod_{m=1}^{M} \prod_{d=1}^{D_m} \mathcal{G}\left(\tau_d^m \,|\, a_0^\tau, b_0^\tau\right).
\end{aligned}
\tag{10}
$$

This completes the definition of the model, which is graphically illustrated in **Appendix Figure S24**.

3 Model Inference

3.1 Introduction to variational Bayes inference

To ensure scalable inference we use a variational approach with a mean-field approximation[3]. Briefly, in variational inference the true intractable posterior distribution of the unobserved variables $p(\mathbf{X}|\mathbf{Y})$ is approximated by a simpler distribution of factorized form $q(\mathbf{X}) = \prod_i q(\mathbf{X}_i)$ that leads to an efficient inference scheme. Here, $\mathbf{X}$ denotes all the hidden variables (including parameters) and $\mathbf{Y}$ denotes all the observed variables.
Under this approximation, the true log marginal likelihood $\log p(\mathbf{Y})$ is lower bounded by:

$$
\begin{aligned}
\mathcal{L}(\mathbf{X}) &= \int q(\mathbf{X}) \Big(\log \frac{p(\mathbf{X}|\mathbf{Y})}{q(\mathbf{X})} + \log p(\mathbf{Y}) \Big) d\mathbf{X} \\
&= \log p(\mathbf{Y}) - \text{KL}(q(\mathbf{X})||p(\mathbf{X}|\mathbf{Y})) \\
&\leq \log p(\mathbf{Y})
\end{aligned}
\tag{11}
$$

$\mathcal{L}(\mathbf{X})$ is called the Evidence Lower Bound (ELBO), which is equal to the sum of the model evidence and the negative KL-divergence between the true posterior and the variational distribution. The key observation here is that increasing the ELBO is equivalent to decreasing the KL-divergence between the two distributions.
Variational learning involves optimising the functional $\mathcal{L}(\mathbf{X})$ with respect to the distribution $q(\mathbf{X})$. If we allow any possible choice of $q(\mathbf{X})$, then the maximum of the lower bound $\mathcal{L}(\mathbf{X})$ will occur when the KL-divergence vanishes, which occurs when $q(\mathbf{X})$ equals the true posterior distribution $p(\mathbf{X}|\mathbf{Y})$. Nevertheless, since the true posterior is intractable, this does not lead to any simplification of the problem. Instead, it is necessary to consider a restricted family of variational distributions that are tractable to compute and then seek the member of this family for which the KL divergence is minimised [2].

Mean-field approximation

The most common type of variational Bayes, known as mean-field approach, assumes that the variational distribution factorises over M disjoint groups of variables:

$$
q(\mathbf{X}) = \prod_{i=1}^{M} q(\mathbf{x}_i)
$$

Evidently, this family of distributions does not usually contain the true posterior because the unobserved variables have dependencies, but this assumption allows the derivation of an analytical inference scheme

[2].
It follows that the optimal distribution $\hat{q}_i$ that maximises the lower bound $\mathcal{L}(\mathbf{X})$, for each variable $\mathbf{x}_i$, can be calculated as follows:

$$\log \hat{q}_i(\mathbf{x}_i) = \mathbb{E}_{-i}[\log p(\mathbf{Y}, \mathbf{X})] + \text{const} \tag{12}$$

where $\mathbb{E}_{-i}$ denotes an expectation with respect to the q distributions over all variables $\mathbf{x}_j$ except for $\mathbf{x}_i$. The additive constant is set by normalising the distribution $\hat{q}_i(\mathbf{z}_i)$:

$$\hat{q}_i(\mathbf{x}_i) = \frac{\exp(\mathbb{E}_{-i}[\log p(\mathbf{Y}, \mathbf{X})])}{\int \exp(\mathbb{E}_{-i}[\log p(\mathbf{Y}, \mathbf{X})]) d\mathbf{X}}$$

This is the general expression which yields the set of variational distributions that maximise the lower bound of the log marginal likelihood, subject to the factorisation constraint. Or equivalently, the set of distributions that minimise the KL divergence between the $q(\mathbf{X})$ distribution and the true posterior $p(\mathbf{X})$.
For MOFA we adopt the following mean field approximation, which factorizes in all model variables except for $\hat{w}^m_{d,k}, s^m_{d,k}$, which are strongly connected by the re-parametrization $w^m_{d,k} = \hat{w}^m_{d,k} s^m_{d,k}$:

$$\begin{aligned} q(\mathbf{Z}, \mathbf{S}, \hat{\mathbf{W}}, \boldsymbol{\alpha}, \boldsymbol{\tau}, \boldsymbol{\theta}) &= q(\mathbf{Z}) q(\boldsymbol{\alpha}) q(\boldsymbol{\theta}) q(\boldsymbol{\tau}) q(\mathbf{S}, \hat{\mathbf{W}}) \\ &= \prod_{n=1}^{N} \prod_{k=1}^{K} q(z_{n,k}) \prod_{m=1}^{M} \prod_{k=1}^{K} q(\alpha^m_k) q(\theta^m_k) \prod_{m=1}^{M} \prod_{d=1}^{D_m} q(\tau^m_d) \prod_{m=1}^{M} \prod_{d=1}^{D_m} \prod_{k=1}^{K} q(\hat{w}^m_{d,k}, s^m_{d,k}) \end{aligned}$$

Variational Bayes expectation maximization algorithm

Note that in Equation (12), for a given variable $\mathbf{x}_i$, the expectation on the right-hand side is taken with respect to the other variables' variational distribution $q_j(\mathbf{x}_j)$ for $j \neq i$. Therefore, there are circular dependencies between the different equations and there is no analytical solution for the parameters of the variational distribution. This naturally suggests an iterative algorithm similar to the Expectation Maximisation (EM) algorithm. In each step we update the moments and parameters of the variational distribution of the latent variables $q_j(\mathbf{x}_j)$ using the current estimates of the variational distributions of the parameters $q_{-j}(\mathbf{x}_{-j})$ [2]. The algorithm is stopped when the change in the ELBO is small enough.

3.2 Update equations for Gaussian data

Latent variables

Variational distribution:

$$q(\mathbf{Z}) = \prod_{k=1}^{K} \prod_{n=1}^{N} q(z_{nk}) = \prod_{k=1}^{K} \prod_{n=1}^{N} \mathcal{N}(z_{nk} \mid \mu_{z_{nk}}, \sigma_{z_{nk}})$$

where

$$\begin{aligned} \sigma^2_{z_{nk}} &= \Big(\sum_{m=1}^{M} \sum_{d=1}^{D_m} \langle \tau^m_d \rangle \langle (s^m_{dk} \hat{w}^m_{dk})^2 \rangle + 1 \Big)^{-1} \\ \mu_{z_{nk}} &= \sigma^2_{z_{nk}} \sum_{m=1}^{M} \sum_{d=1}^{D_m} \langle \tau^m_d \rangle \langle s^m_{dk} \hat{w}^m_{dk} \rangle \Big(y^m_{nd} - \sum_{j \neq k} \langle s^m_{dj} \hat{w}^m_{dj} \rangle \langle z_{nj} \rangle \Big) \end{aligned}$$

Spike and Slab weights

Variational distribution:

$$q(\hat{\mathbf{W}}, \mathbf{S}) = \prod_{m=1}^{M} \prod_{d=1}^{D_m} \prod_{k=1}^{K} q(\hat{w}^m_{dk}, s^m_{dk}) = \prod_{m=1}^{M} \prod_{d=1}^{D_m} \prod_{k=1}^{K} q(\hat{w}^m_{dk} | s^m_{dk}) q(s^m_{dk})$$

Update for $q(s^m_{dk})$:

$$\gamma_{dk^m} = q(s_{dk} = 1) = \frac{1}{1 + \exp(-\lambda_{dk^m})},$$

where

$$\lambda_{dk}^m = \langle \log \frac{\theta}{1-\theta} \rangle + 0.5 \log \frac{\langle \alpha_k^m \rangle}{\langle \tau_d^m \rangle} - 0.5 \log \Big(\sum_{n=1}^N \langle z_{nk}^2 \rangle + \frac{\langle \alpha_k^m \rangle}{\langle \tau_d^m \rangle} \Big) + \frac{\langle \tau_d^m \rangle}{2} \frac{\Big(\sum_{n=1}^N y_{nd}^m \langle z_{nk} \rangle - \sum_{j \neq k} \langle s_{dj}^m \hat{w}_{dj}^m \rangle \sum_{n=1}^N \langle z_{nk} \rangle \langle z_{nj} \rangle \Big)^2}{\sum_{n=1}^N \langle z_{nk}^2 \rangle + \frac{\langle \alpha_k^m \rangle}{\langle \tau_d^m \rangle}}$$

Update for $q(\hat{w}_{dk}^m)$:

$$q(\hat{w}_{dk}^m | s_{dk}^m = 0) = \mathcal{N}\left(\hat{w}_{dk}^m \,|\, 0, 1/\alpha_k^m\right),$$
$$q(\hat{w}_{dk}^m | s_{dk}^m = 1) = \mathcal{N}\left(\hat{w}_{dk}^m \,|\, \mu_{w_{dk}^m}, \sigma_{w_{dk}^m}^2\right),$$

where

$$\mu_{w_{dk}^m} = \frac{\sum_{n=1}^N y_{nd}^m \langle z_{nk} \rangle - \sum_{j \neq k} \langle s_{dj}^m \hat{w}_{dj}^m \rangle \sum_{n=1}^N \langle z_{nk} \rangle \langle z_{nj} \rangle}{\sum_{n=1}^N \langle z_{nk}^2 \rangle + \frac{\langle \alpha_k^m \rangle}{\langle \tau_d^m \rangle}}$$
$$\sigma_{w_{dk}^m} = \frac{\langle \tau_d^m \rangle^{-1}}{\sum_{n=1}^N \langle z_{nk}^2 \rangle + \frac{\langle \alpha_k^m \rangle}{\langle \tau_d^m \rangle}}$$

Taken together this means that we can update $q(\hat{w}_{dk}^m, s_{dk}^m)$ using:

$$q(\hat{w}_{dk}^m | s_{dk}^m) q(s_{dk}^m) = \mathcal{N}\left(\hat{w}_{dk}^m \,|\, s_{dk}^m \mu_{w_{dk}^m}, s_{dk}^m \sigma_{w_{dk}^m}^2 + (1 - s_{dk}^m)/\alpha_k^m\right) (\gamma_{dk}^m)^{s_{dk}^m} (1 - \gamma_{dk}^m)^{1 - s_{dk}}$$

ARD precision (alpha)

Variational distribution:

$$q(\boldsymbol{\alpha}) = \prod_{m=1}^M \prod_{k=1}^K \mathcal{G}(\alpha_k^m | \hat{a}_{mk}^\alpha, \hat{b}_{mk}^\alpha)$$

where

$$\hat{a}_{mk}^\alpha = a_0^\alpha + \frac{D_m}{2}$$
$$\hat{b}_{mk}^\alpha = b_0^\alpha + \frac{\sum_{d=1}^{D_m} \langle (\hat{w}_{d,k}^m)^2 \rangle}{2}$$

Noise precision (tau)

Variational distribution:

$$q(\boldsymbol{\tau}) = \prod_{m=1}^M \prod_{d=1}^{D_m} q(\tau_d^m) = \prod_{m=1}^M \prod_{d=1}^{D_m} \mathcal{G}(\tau_d^m | \hat{a}_{md}^\tau, \hat{b}_{md}^\tau)$$

where

$$\hat{a}_{md}^\tau = a_0^\tau + \frac{N}{2}$$
$$\hat{b}_{md}^\tau = b_0^\tau + \frac{1}{2} \sum_{n=1}^N \langle (y_{nd}^m - \sum_k^K \hat{w}_{dk}^m s_{dk}^m z_{n,k})^2 \rangle$$

Spike and Slab sparsity parameter (theta)

Variational distribution:

$$q(\theta) = \prod_{m=1}^M \prod_{k=1}^K \text{Beta}(\theta_k^m | \hat{a}_{mk}^\theta, \hat{b}_{mk}^\theta),$$

where

$$\hat{a}^{\theta}_{mk} = \sum_{d=1}^{D_m} \langle s^m_{dk} \rangle + a^{\theta}_0$$

$$\hat{b}^{\theta}_{mk} = b^{\theta}_0 - \sum_{d=1}^{D_m} \langle s^m_{dk} \rangle + D_m$$

Evidence Lower bound

In order to monitor training and assess convergence we calculate the ELBO alongside with the other updates. The ELBO can be decomposed into a likelihood term and terms for each model variable $\mathbf{X}_i$:

$$\begin{aligned}\mathcal{L}(\mathbf{X}) &= \int q(\mathbf{X})\Big(\log \frac{p(\mathbf{X},\mathbf{Y})}{q(\mathbf{X})}\Big)d\mathbf{X} \\ &= \mathbb{E}_q \log p(\mathbf{Y}|\mathbf{X})\rangle + \sum_i \left(\mathbb{E}_q \log p(\mathbf{X}_i) - \mathbb{E}_q \log q(\mathbf{X}_i)\right),\end{aligned}$$

where the expectation is under the variational distribution of the current step. Each of the terms from the last term is computed as follows:

Likelihood term

If using the gaussian likelihood:

$$-\sum_{m=1}^{M} \frac{ND_m}{2}\log(2\pi) + \frac{N}{2}\sum_{m=1}^{M}\sum_{d=1}^{D_m}\log(\langle \tau^m_d \rangle) - \sum_{m=1}^{M}\sum_{d=1}^{D_m}\frac{\langle \tau^m_d \rangle}{2}\sum_{n=1}^{N}\big(y^m_{nd} - \sum_{k=1}^{K}\langle s^m_{dk}\hat{w}^m_{dk}\rangle\langle z_{nk}\rangle\big)^2$$

When not using the gaussian model, this expression is replaced by the corresponding likelihood.

W and S terms

$$\begin{aligned}\mathbb{E}_q[\log p(\hat{\mathbf{W}},\mathbf{S})] = &-\sum_{m=1}^{M}\frac{KD_m}{2}\log(2\pi) + \sum_{m=1}^{M}\frac{D_m}{2}\sum_{k=1}^{K}\log(\alpha^m_k) - \sum_{m=1}^{M}\frac{\alpha^m_k}{2}\sum_{d=1}^{D_m}\sum_{k=1}^{K}\langle(\hat{w}^m_{dk})^2\rangle \\ &+ \langle\log(\theta)\rangle\sum_{m=1}^{M}\sum_{d=1}^{D_m}\sum_{k=1}^{K}\langle s^m_{dk}\rangle + \langle\log(1-\theta)\rangle\sum_{m=1}^{M}\sum_{d=1}^{D_m}\sum_{k=1}^{K}(1-\langle s^m_{dk}\rangle)\end{aligned}$$

$$\begin{aligned}\mathbb{E}_q[\log q(\hat{\mathbf{W}},\mathbf{S})] = &-\sum_{m=1}^{M}\frac{KD_m}{2}\log(2\pi) + \frac{1}{2}\sum_{m=1}^{M}\sum_{d=1}^{D_m}\sum_{k=1}^{K}\log(\langle s^m_{dk}\rangle\sigma^2_{w^m_{dk}} + (1-\langle s^m_{dk}\rangle)/\alpha^m_k) \\ &+ \sum_{m=1}^{M}\sum_{d=1}^{D_m}\sum_{k=1}^{K}(1-\langle s^m_{dk}\rangle)\log(1-\langle s^m_{dk}\rangle) - \langle s^m_{dk}\rangle\log\langle s^m_{dk}\rangle\end{aligned}$$

Z term

$$\mathbb{E}_q[\log p(\mathbf{Z})] = -\frac{NK}{2}\log(2\pi) - \frac{1}{2}\sum_{n=1}^{N}\langle z^2_{nk}\rangle$$

$$\mathbb{E}_q[\log q(\mathbf{Z})] = -\frac{NK}{2}(1+\log(2\pi)) - \frac{1}{2}\sum_{n=1}^{N}\sum_{k=1}^{K}\log(\sigma^2_{z_{nk}})$$

alpha term

$$\mathbb{E}_q[\log p(\boldsymbol{\alpha})] = \sum_{m=1}^{M}\sum_{k=1}^{K}\Big(a^{\alpha}_0\log b^{\alpha}_0 + (a^{\alpha}_0 - 1)\langle\log\alpha_k\rangle - b^{\alpha}_0\langle\alpha_k\rangle - \log\Gamma(a^{\alpha}_0)\Big)$$

$$\mathbb{E}_q[\log q(\boldsymbol{\alpha})] = \sum_{m=1}^{M}\sum_{k=1}^{K}\Big(\hat{a}^{\alpha}_k\log\hat{b}^{\alpha}_k + (\hat{a}^{\alpha}_k - 1)\langle\log\alpha_k\rangle - \hat{b}^{\alpha}_k\langle\alpha_k\rangle - \log\Gamma(\hat{a}^{\alpha}_k)\Big)$$

tau term

$$\mathbb{E}_q[\log p(\tau)] = \sum_{m=1}^{M} D_m a_0^\tau \log b_0^\tau + \sum_{m=1}^{M}\sum_{d=1}^{Dm}(a_0^\tau - 1)\langle \log \tau_d^m \rangle - \sum_{m=1}^{M}\sum_{d=1}^{Dm} b_0^\tau \langle \tau_d^m \rangle - \sum_{m=1}^{M} D_m \log\Gamma(a_0^\tau)$$

$$\mathbb{E}_q[\log q(\boldsymbol{\tau})] = \sum_{m=1}^{M}\sum_{d=1}^{D_m}\Big(\hat{a}_{dm}^\tau \log \hat{b}_{dm}^\tau + (\hat{a}_{dm}^\tau - 1)\langle \log \tau_d^m \rangle - \hat{b}_{dm}^\tau \langle \tau_d^m \rangle - \log\,\Gamma(\hat{a}_{dm}^\tau)\Big)$$

theta term

$$\mathbb{E}_q\left[\log p(\theta)\right] = \sum_{m=1}^{M}\sum_{k=1}^{K}\sum_{d=1}^{D_m}\big((a_0 - 1) \times \langle \log(\pi_{d,k}^m)\rangle + (b_0 - 1)\langle \log(1 - \pi_{d,k}^m)\rangle - \log(\mathrm{B}(a_0, b_0))\big)$$

$$\mathbb{E}_q\left[\log q(\theta)\right] = \sum_{m=1}^{M}\sum_{k=1}^{K}\sum_{d=1}^{D_m}\big((a_{k,d}^m - 1) \times \langle \log(\pi_{d,k}^m)\rangle + (b_{k,d}^m - 1)\langle \log(1 - \pi_{d,k}^m)\rangle - \log(\mathrm{B}(a_{k,d}^m, b_{k,d}^m))\big)$$

4 Modelling and inference with non-Gaussian data

To implement efficient variational inference in conjunction with a non-Gaussian likelihood we adapt prior work from [11] using local variational bounds. The key idea is to dynamically approximate non-Gaussian data by Gaussian pseudo-data based on a second-order Taylor expansion. To make the approximation justifiable we need to introduce variational parameters that are adjusted alongside the updates to improve the fit.

Denoting the parameters in the MOFA model as $\mathbf{X} = (\mathbf{Z}, \mathbf{W}, \boldsymbol{\alpha}, \boldsymbol{\tau}, \boldsymbol{\theta})$, recall that the variational framework approximates the posterior $p(\mathbf{X}|\mathbf{Y})$ with a distribution $q(\mathbf{X})$, which is indirectly optimised by optimising a lower bound of the log model evidence. The resulting optimization problem can be re-written from Equation (11) as

$$\min_{q(\mathbf{X})} -\mathcal{L}(\mathbf{X}) = \min_{q(\mathbf{X})} \mathbb{E}_q\big[-\log p(\mathbf{Y}|\mathbf{X})\big] + \mathrm{KL}[q(\mathbf{X})||p(\mathbf{X})].$$

Expanding the MOFA model to non-Gaussian likelihoods we now assume a general likelihood of the form $p(\mathbf{Y}|\mathbf{X}) = p(\mathbf{Y}|\mathbf{C})$ with $\mathbf{C} = \mathbf{Z}\mathbf{W}^T$, that can write as

$$-\log p(\mathbf{Y}|\mathbf{X}) = \sum_{n=1}^{N}\sum_{d=1}^{D} f_{nd}(c_{nd})$$

with $f_{nd}(c_{nd}) = -\log p(y_{nd}|c_{nd})$. We dropped the view index m to keep notation uncluttered.

Extending [11] to our heteroscedastic noise model, we require $f_{nd}(c_{nd})$ to be twice differentiable and bounded by κ_d, such that $f''_{nd}(c_{nd}) \leq \kappa_d\, \forall n, d$. This holds true in many important models as for example the Bernoulli and Poisson case. Under this assumption a lower bound on the log likelihood can be constructed using Taylor expansion,

$$f_{nd}(c_{nd}) \leq \frac{\kappa_d}{2}(c_{nd} - \zeta_{nd})^2 + f'(\zeta_{nd})(c_{nd} - \zeta_{nd}) + f_{nd}(\zeta_{nd}) := q_{nd}(c_{nd}, \zeta_{nd}),$$

where $\boldsymbol{\zeta} = \zeta_{nd}$ are additional variational parameters that determine the location of the Taylor expansion and have to be optimised to make the lower bound as tight as possible. Plugging the bounds into above optimization problem, we obtain:

$$\min_{q(\mathbf{X}),\boldsymbol{\zeta}} \quad \sum_{d=1}^{D}\sum_{n=1}^{N} \mathbb{E}_q[q_{nd}(c_{nd}, \zeta_{nd})] + \mathrm{KL}[q(\mathbf{X})||p(\mathbf{X})]$$

The algorithm propsed in [11] then alternates between updates of $\boldsymbol{\zeta}$ and q($\boldsymbol{\Theta}$). The update for $\boldsymbol{\zeta}$ is given by

$$\zeta \leftarrow \mathbb{E}[\mathbf{W}]\mathbb{E}[\mathbf{Z}]^T$$

where the expctations are taken with respect to the corresponding q distributions.

On the other hand, the updates for $q(\mathbf{X})$ can be shown to be identical to the variational Bayesian updates with a conjugate Gaussian likelihood when replacing the observed data $\mathbf{Y}$ by a pseudo-data $\hat{\mathbf{Y}}$ and the

precisions τ_{nd} (which were treated as random variables) by the constant terms κ_d introduced above. The pseudodata is given by

$$\hat{y}_{nd} = \zeta_{nd} - f'(\zeta_{nd})/\kappa_d.$$

Depending on the log likelihoods $f(\cdot)$ different κ_d are used resulting in different pseudo-data updates. Two special cases implemented in MOFA are the Poisson and Bernoulli likelihood described in the following.

Bernoulli likelihood for binary data

When the observations are binary, $y \in \{0, 1\}$, they can be modelled using a Bernoulli likelihood:

$$\mathbf{Y}|\mathbf{Z}, \mathbf{W} \sim \text{Ber}(\sigma(\mathbf{Z}\mathbf{W}^T)),$$

where $\sigma(a) = (1 + e^{-a})^{-1}$ is the logistic link function and $\mathbf{Z}$ and $\mathbf{W}$ are the latent factors and weights in our model, respectively.
In order to make the variational inference efficient and explicit as in the Gaussian case, we aim to approximate the Bernoulli data by a Gaussian pseudo-data as proposed in [11] and described above which allows to recycle all the updates from the model with Gaussian views. While [11] assumes a homoscedastic approximation with a spherical Gaussian, we adopt an approach following [6], which allows for heteroscedaticity and provides a tighter bound on the Bernoulli likelihood.
Denoting $c_{nd} = (\mathbf{Z}\mathbf{W}^T)_{nd}$ the Jaakkola upper bound [6] on the negative log-likelihood is given by

$$\begin{aligned}
-\log\left(p(y_{nd}|c_{nd})\right) &= -\log\left(\sigma\left((2y_{nd} - 1)c_{nd}\right)\right) \\
&\leq -\log(\zeta_{nd}) - \frac{(2y_{nd} - 1)c_{nd} - \zeta_{nd})}{2} + \lambda(\zeta_{nd})\left(c_{nd}^2 - \zeta_{nd}^2\right) \\
&=: b_J(\zeta_{nd}, c_{nd}, y_{nd})
\end{aligned}$$

with λ given by $\lambda(\zeta) = \frac{1}{4\zeta}\tanh\left(\frac{\zeta}{2}\right)$.
This can easily be derived from a first-order Taylor expansion on the function $f(x) = -\log(e^{\frac{x}{2}} + e^{-\frac{x}{2}}) = \frac{x}{2} - \log(\sigma(x))$ in x^2 and by the convexity of f in x^2 this bound is global as discussed in [6].
In order to make use of this tighter bound but still be able to re-use the variational updates from the Gaussian case we re-formulate the bound as a Gaussian likelihood on pseudo-data $\hat{\mathbf{Y}}$.
As above we can plug this bound on the negative log-likelihood into the variational optimization problem to obtain

$$\min_{q(\mathbf{X}), \boldsymbol{\zeta}} \sum_{d=1}^{D}\sum_{n=1}^{N} \mathbb{E}_q b_J(\zeta_{nd}, c_{nd}, y_{nd}) + \text{KL}[q(\mathbf{X})||p(\mathbf{X})].$$

This is minimized iteratively in the variational parameter ζ_{nd} and the variational distribution of Z,W: Minimizing in the variational parameter ζ this leads to the updates given by

$$\zeta_{nd}^2 = \mathbb{E}[c_{nd}^2]$$

as described in [6], [3].
For the variational distribution $q(\mathbf{Z}, \mathbf{W})$ we observe that the Jaakkola bound can be re-written as

$$b_J(\zeta_{nd}, c_{nd}, y_{nd}) = -\log\left(\varphi\left(\hat{y}_{nd}; c_{nd}, \frac{1}{2\lambda(\zeta_{nd})}\right)\right) + \gamma(\zeta_{nd}),$$

where $\varphi(\cdot; \mu, \sigma^2)$ denotes the density function of a normal distribution with mean μ and variance σ^2 and γ is a term only depending on $\boldsymbol{\zeta}$. This allows us to re-use the updates for $\mathbf{Z}$ and $\mathbf{W}$ from a setting with Gaussian likelihood by considering the Gaussian pseudo-data

$$\hat{y}_{nd} = \frac{2y_{nd} - 1}{4\lambda(\zeta_{nd})}$$

updating the data precision as $\tau_{nd} = 2\lambda(\zeta_{nd})$ using updates generalized for sample- and feature-wise precision parameters on the data.

Poisson likelihood for count data

When observations are a natural numbers, such as count data $y \in \mathbb{N} = \{0, 1, \cdots\}$, they can be modelled using a Poisson likelihood:

$$p(y|c) = \lambda(c)^y e^{-\lambda(c)}$$

where $\lambda(c) > 0$ is the rate function and has to be convex and log-concave in order to ensure that the likelihood is log-concave.
As done in [11], here we choose the following rate function: $\lambda(c) = \log(1 + e^c)$.
Then an upper bound of the second derivative of the log-likelihood is given by

$$f''_{nd}(c_{nd}) \leq \kappa_d = 1/4 + 0.17 * \max(\mathbf{y}_{:,d}).$$

The pseudodata updates are given by

$$\hat{y}_{nd} = \zeta_{nd} - \frac{\mathrm{S}(\zeta_{nd})(1 - y_{nd}/\lambda(\zeta_{nd}))}{\kappa_d}.$$

5 Implementation and practical considerations for training

5.1 Monitoring convergence

In contrast to sampling methods, variational approximations have the appealing property that convergence is easily monitored by changes in the ELBO, which is required to increase monotonically [3]. In practice, we set a default threshold for convergence corresponding to a change in ELBO smaller than 0.1%.

5.2 Handling of missing values

The model naturally accounts for missing values and no prior imputation is required. Non-observed data points do not intervene in the likelihood and are ignored in the update equations. In practice, we use a binary mask $\mathcal{O}^m \in \mathbb{R}^{N \times D_m}$ for each view m, such that $\mathcal{O}_{n,d} = 1$ when feature d is observed for sample n, 0 otherwise.

5.3 Data pre-processing

MOFA does not require the data to be centered or scaled. The first property is achieved by incorporating a constant factor of ones that will capture any feature-wise intercept effect. This ensures that the rest of the factors capture variation independent of the feature-wise means. The second property is achieved by the factor- and view-wise ARD prior, which allows different scales of the weights for each view.
However, when using the Gaussian noise model, it is recommended to use methods for normalization and variance stabilisation (e.g. as implemented in [8] for RNAseq data) prior to model training. This ensures that the normality assumption of the model residuals is appropriate.

5.4 Consistency across random initilizations

The variational Bayes algorithm is not guaranteed to find the optimal solution [3] and the estimates will depend on the parameter initialization. We suggest to adopt common practice [5] and assess the consistency of factors by running MOFA multiple times (e.g. 10 trials) under different initialisations. Subsequently, a single model with the highest ELBO should be selected for downstream analysis.
Appropriate functions for model selection are provided in the R package.

5.5 Determining the number of factors

The model can automatically learn the number of factors by removing inactive factors during training if they do not explain significant variation in any view. This is achieved by the view- and factor-wise ARD prior (Eq. (7)). In practice, factors are pruned during training using a minimum fraction of variance explained threshold that needs to be specified by the user. Alternatively, the user can fix the number of factors and the minimum variance criterion is ignored.

5.6 Rotational invariance

An important consequence of the definition of MOFA (and most factor analysis models [1, 13]) is their unidentifiability due to rotational and scaling invariance. This means that the factors and corresponding loadings can only be identified up to an orthogonal rotation. In practice, this property implies that the actual factor and weight values need to be interpreted in a relative manner, always within the same model instance.

References

[1] A. T. Basilevsky. *Statistical factor analysis and related methods: theory and applications.* Vol. 418. John Wiley & Sons, 2009.

[2] J. Beal. "Variational algorithms for approximate bayesian inference". University College London, 2003.

[3] C. M. Bishop. "Pattern recognition". In: *Machine Learning* 128 (2006), pp. 1–58.

[4] F. Buettner et al. "f-scLVM: scalable and versatile factor analysis for single-cell RNA-seq". In: *Genome Biology* (in press).

[5] V. Hore et al. "Tensor decomposition for multiple-tissue gene expression experiments". In: *Nature Genetics* 48.9 (2016), pp. 1094–1100.

[6] T. S. Jaakkola and M. I. Jordan. "Bayesian parameter estimation via variational methods". In: *Statistics and Computing* 10.1 (2000), pp. 25–37.

[7] S. A. Khan et al. "Identification of structural features in chemicals associated with cancer drug response: a systematic data-driven analysis". In: *Bioinformatics* 30.17 (2014), pp. i497–i504.

[8] M. I. Love, W. Huber, and S. Anders. "Moderated estimation of fold change and dispersion for RNA-seq data with DESeq2". In: *Genome biology* 15.12 (2014), p. 550.

[9] D. J. MacKay. "Bayesian methods for backpropagation networks". In: *Models of neural networks III.* Springer, 1996, pp. 211–254.

[10] T. J. Mitchell and J. J. Beauchamp. "Bayesian variable selection in linear regression". In: *Journal of the American Statistical Association* 83.404 (1988), pp. 1023–1032.

[11] M. Seeger and G. Bouchard. "Fast variational Bayesian inference for non-conjugate matrix factorization models". In: *Artificial Intelligence and Statistics.* 2012, pp. 1012–1018.

[12] M. K. Titsias and M. Lázaro-Gredilla. "Spike and slab variational inference for multi-task and multiple kernel learning". In: *Advances in neural information processing systems.* 2011, pp. 2339–2347.

[13] S. Virtanen et al. "Bayesian group factor analysis". In: *Artificial Intelligence and Statistics.* 2012, pp. 1269–1277.

6 Supplementary Figures

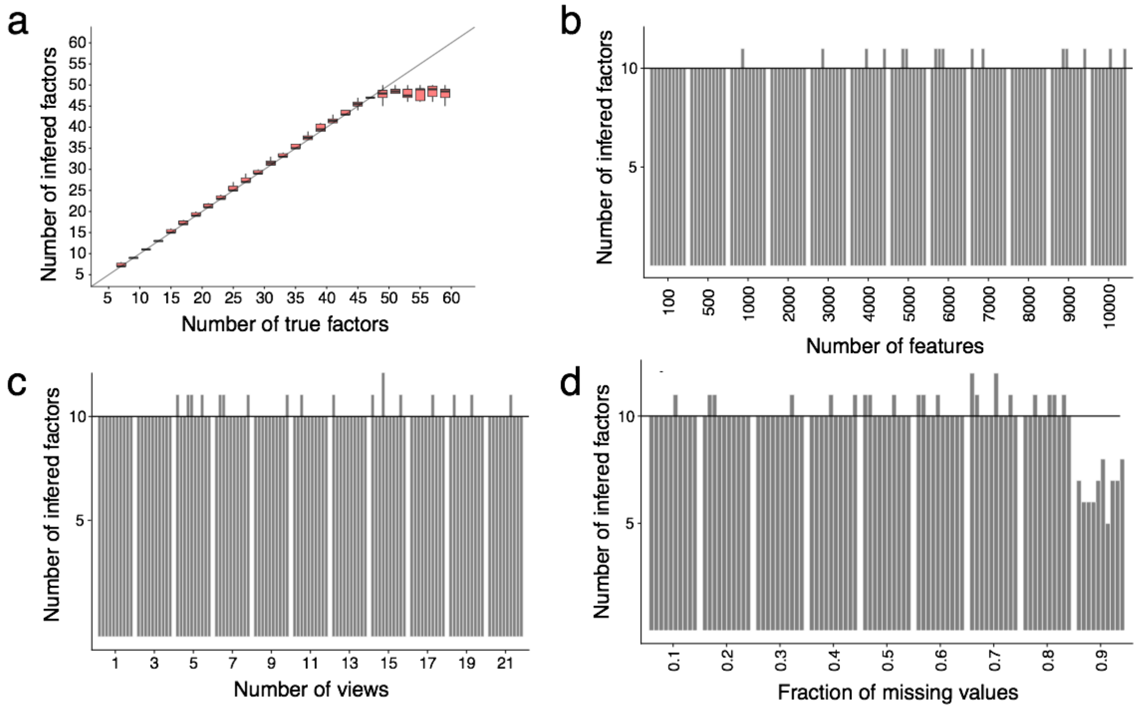

Appendix Figure S1 | Model validation of MOFA using simulated data. (a) Comparison of the number of simulated and estimated factors. Boxplots show the distribution across 10 model instances. **(b-d)** Recovery of the true number of latent factors (*K*=10) under different **(b)** number of features, **(c)** views and **(d)** fraction of missing values. Individual bars correspond to different model instances.

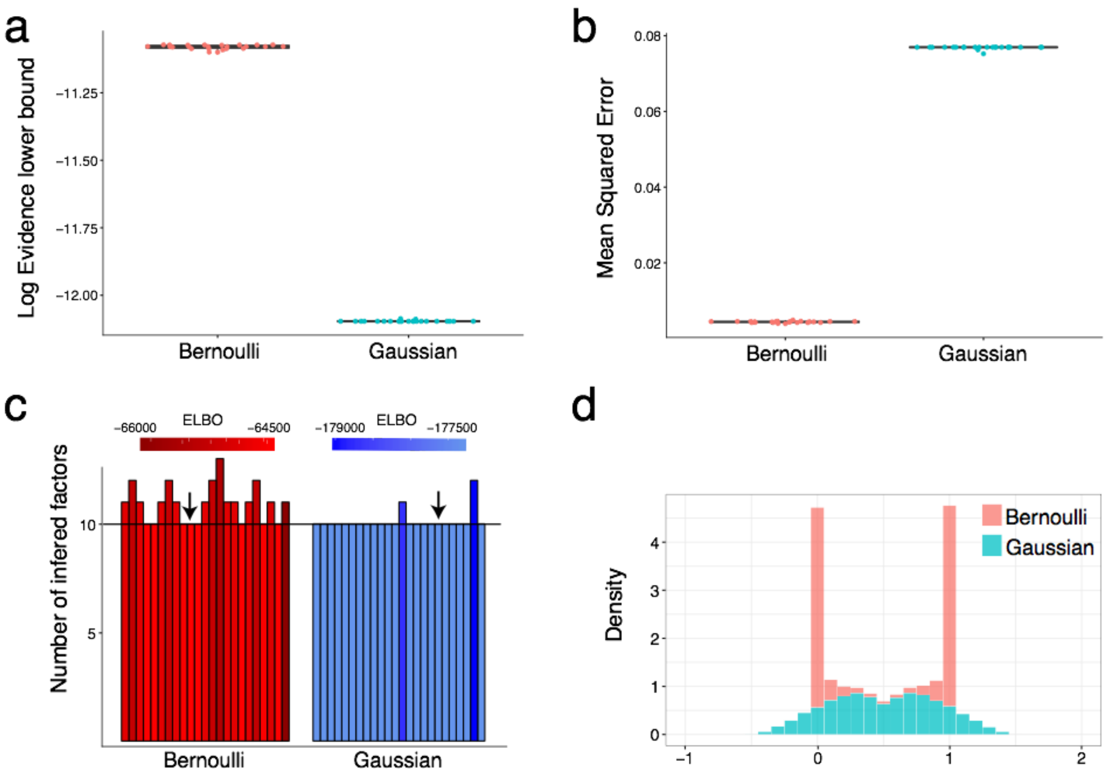

Appendix Figure S2 | Validation of the Bernoulli likelihood model. On a simulated binary data 25 instances of a MOFA model were trained either considering a Bernoulli (red) or Gaussian (blue) likelihood, respectively. **(a)** Variational evidence lower bound (ELBO) for each model instance. **(b)** Reconstruction error for each model instance. **(c)** Number of estimated factors. The horizontal line denotes the true number of factors (K=10). Individual model instances are colored based on their respective ELBO value. The arrows mark the models with the highest ELBO that would be selected for downstream analysis. **(d)** Distribution of the reconstructed data, with the Bernoulli (red) or Gaussian (blue) likelihood model, respectively

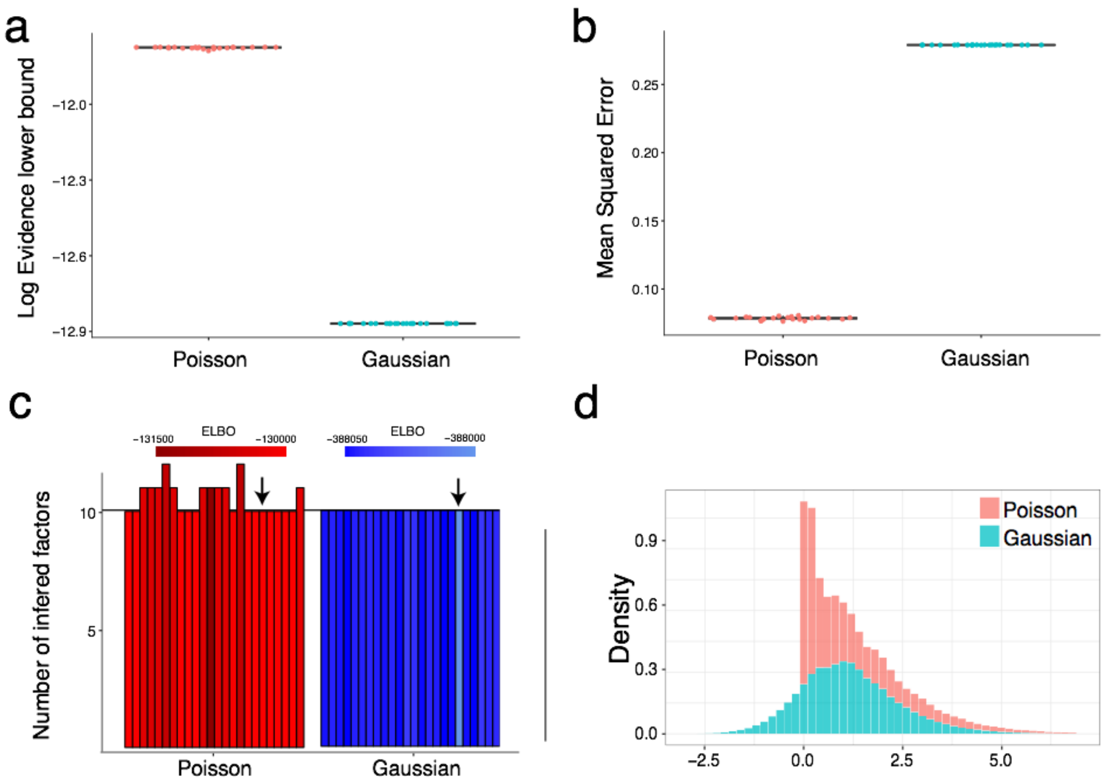

Appendix Figure S3 | Validation of the Poisson likelihood model. On a simulated count data 25 instances of a MOFA model were trained either considering a Poisson (red) or Gaussian (blue) likelihood, respectively. **(a)** Variational evidence lower bound (ELBO) for each model instance. **(b)** Reconstruction error for each model instance. **(c)** Number of estimated factors. The horizontal line denotes the true number of factors (K=10). Individual model instances are colored based on their respective ELBO value. The arrows mark the models with the highest ELBO that would be selected for downstream analysis. **(d)** Distribution of the reconstructed data, with the Poisson (red) or Gaussian (blue) likelihood model, respectively

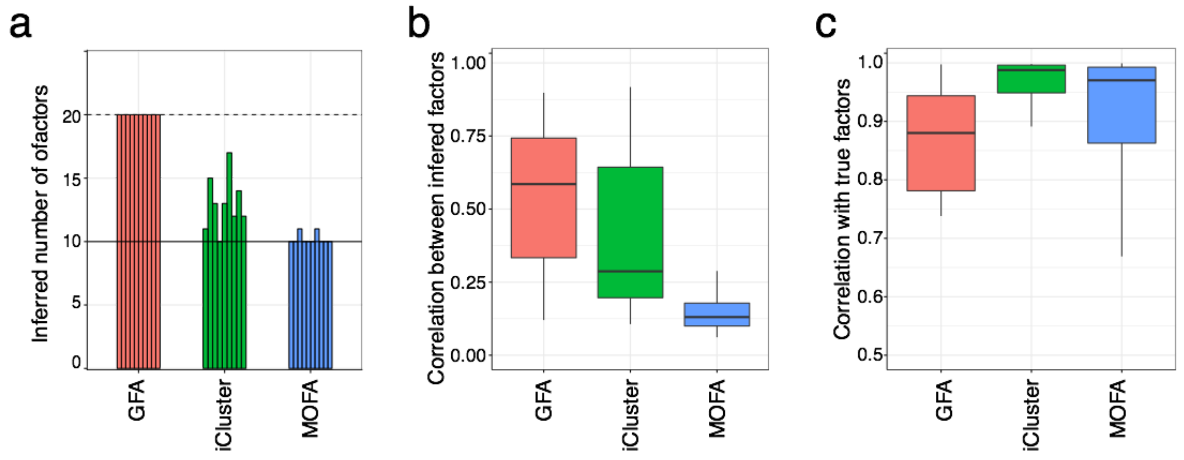

Appendix Figure S4 | Comparison of MOFA, GFA and iCluster on simulated data. (a) Estimated number of factors. The solid horizontal line denotes the true number of simulated factors (*K*=10). and the dashed horizontal line indicates the initial number of factors (*K*=20). Each bar represents a different model realization of the simulated data. **(b)** Pearson correlation coefficient between pairs of inferred latent factors for individual trials. For each factor, shown is the maximum correlation coefficient with any of the remaining factors. Factors were simulated to be uncorrelated. **(c)** Pearson correlation coefficient between true and inferred factors (for the top ten factors in each fit). For each factor, shown is the maximum correlation coefficient with any of the true factors.

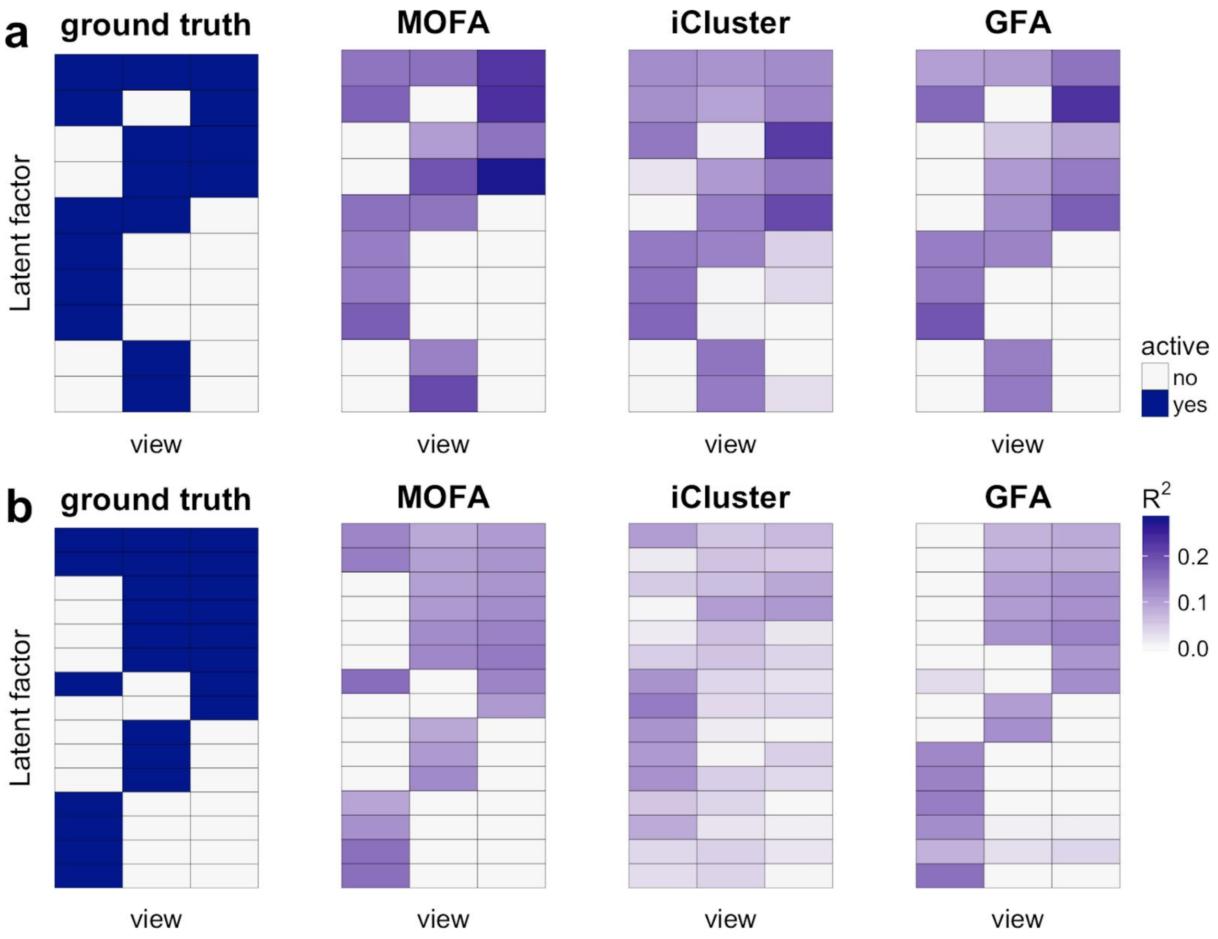

Appendix Figure S5 | Assessment of MOFA, iCluster and GFA in terms of recovering the pattern of factor activity across views. Data were simulated using a Gaussian likelihood based on the true activity pattern of factors per view displayed on the left. **(a)** Number of true underlying factors *K*=10. **(b)** Number of true underlying factor *K*=15. In both cases, MOFA and GFA were trained starting with *K*=25 factors whereas for iCluster the true number of factors was used. Shown is the fraction of variance explained for each factor and view.

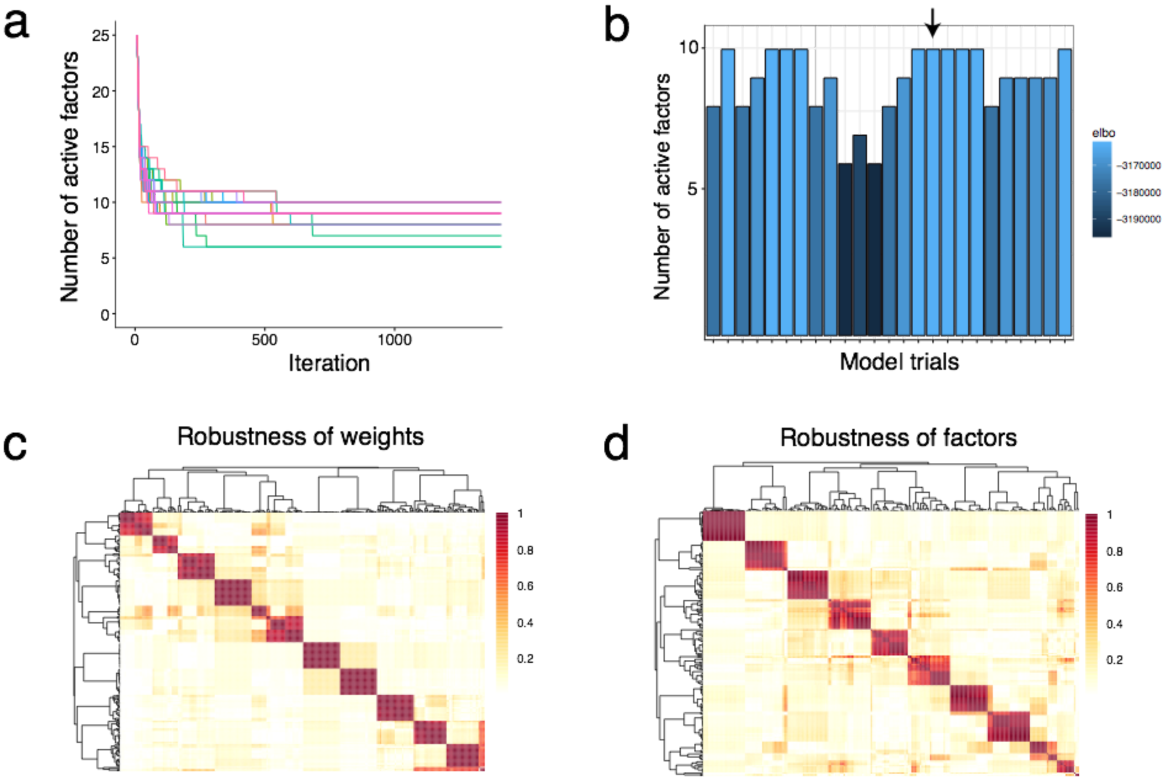

Appendix Figure S6 | Assessment of model consistency across different trials. 25 MOFA instances were trained on the CLL data. **(a)** The training curve for the number of active factors. **(b)** The number of estimated factors for each trial, colored by the corresponding evidence lower bound (ELBO). The arrow indicates the model with highest ELBO that was selected for downstream analysis. **(c)** Absolute value of the Pearson correlation coefficient between the weights of the mRNA data. Each block in the diagonal captures a weight vector consistently learnt across multiple trials. **(d)** Absolute value of the Pearson correlation coefficient between the factors. Each block in the diagonal captures a latent factor consistently learnt across multiple trials.

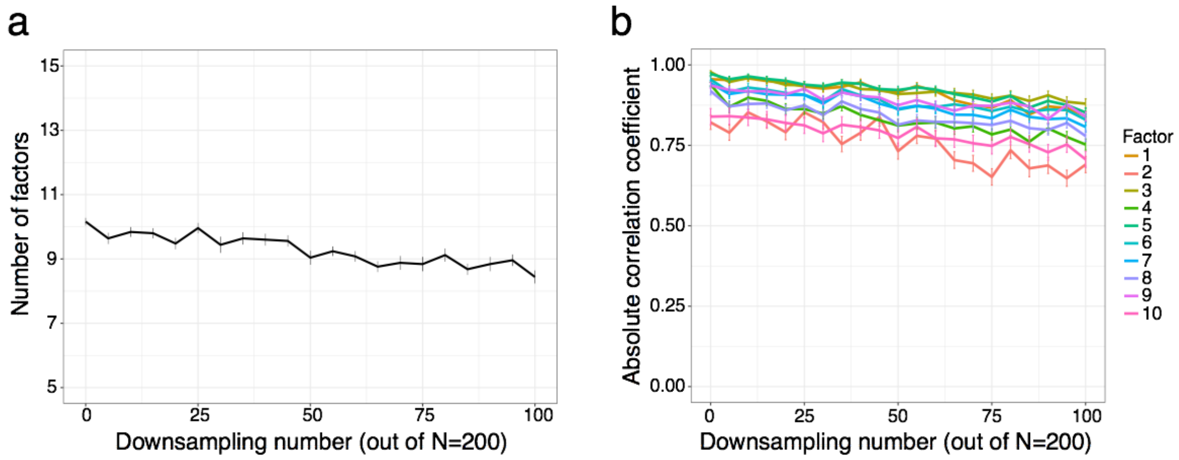

Appendix Figure S7 | Model robustness on the CLL data assessed using downsampling of samples. Shown is **(a)** the number of factors and **(b)** the absolute Pearson correlation coefficient between factors estimated on downsampled data and the factors estimated on the full dataset. Shown are averages across 25 trials. Error bars denote plus or minus one standard error.

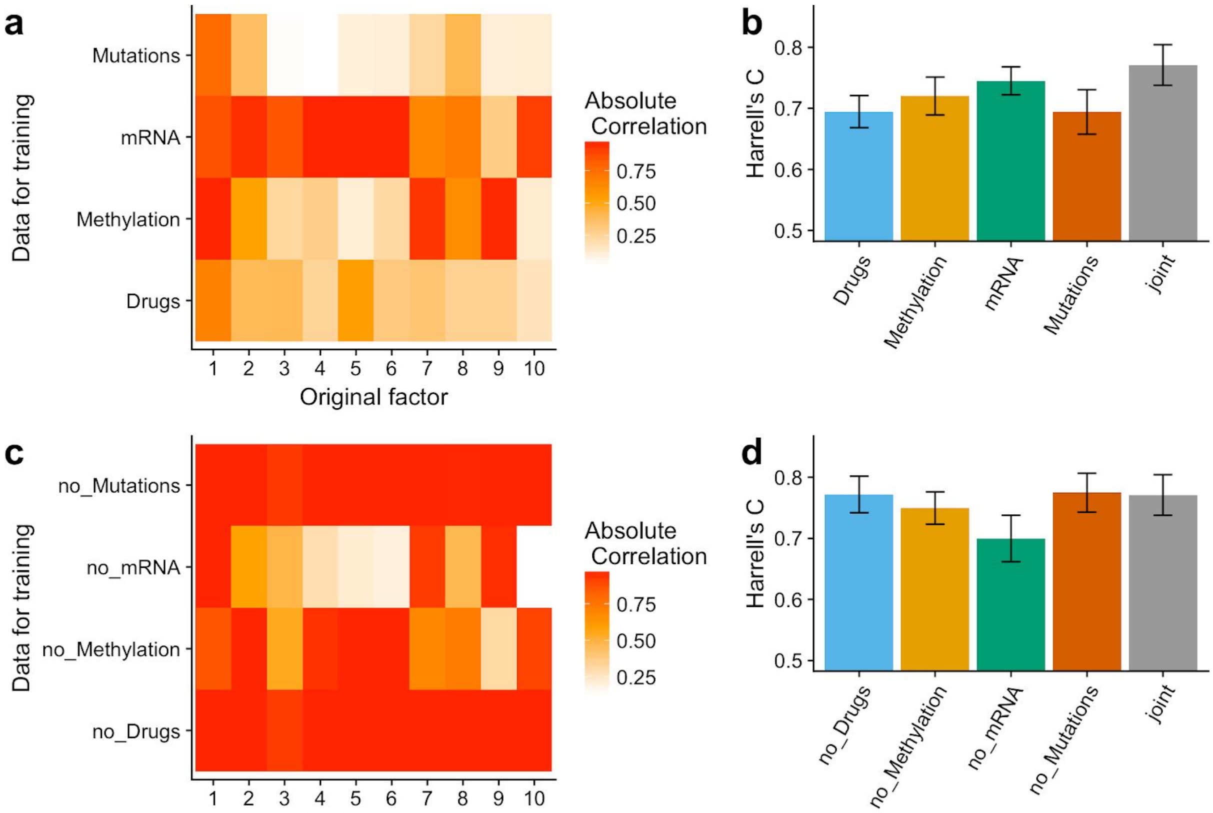

Appendix Figure S8 | MOFA trained on a subset of the available assays. (a) Absolute correlation between the MOFA factors (x-axis) recovered on the full data sets with the most associated factor recovered when using only one data modality (y-axis). Correlation is calculated on the *n*=121 samples that were profiled in all assays. **(b)** Harrell's C-index for prediction of time to next treatment for the *n*=121 samples with data in all modalities using 10 factors obtained using MOFA on each single data modality as well as the full data. **(c)** Same as in **a** for MOFA trained on all assays except one. **(d)** Same as in **b** for MOFA trained on all assays except one.

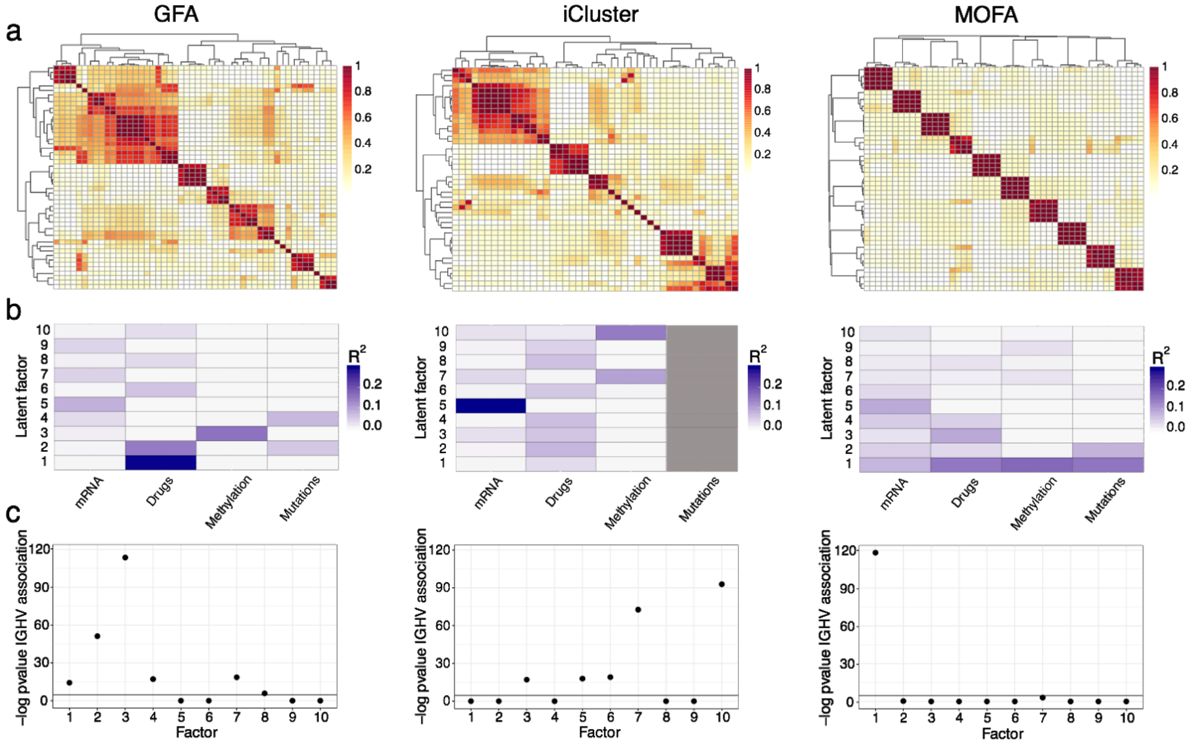

Appendix Figure S9 | Performance of MOFA, GFA and iCluster on CLL data. (a) Consistency of inferred factors across multiple trials. Shown are absolute Pearson correlation coefficient between pairs of factors in different trials. Each diagonal block captures a factor that is consistently learnt across multiple trials. For the first trial of each model, shown is: **(b)** Fraction of variance explained (R^2) by individual factors for each view. No variance measure can be estimated in the (binary) mutation data by the iCluster method. **(c)** Negative log FDR-adjusted p-values from the association analysis (t-test) between individual factors and IGHV status. The line denotes the statistical significance threshold of 1% FDR.

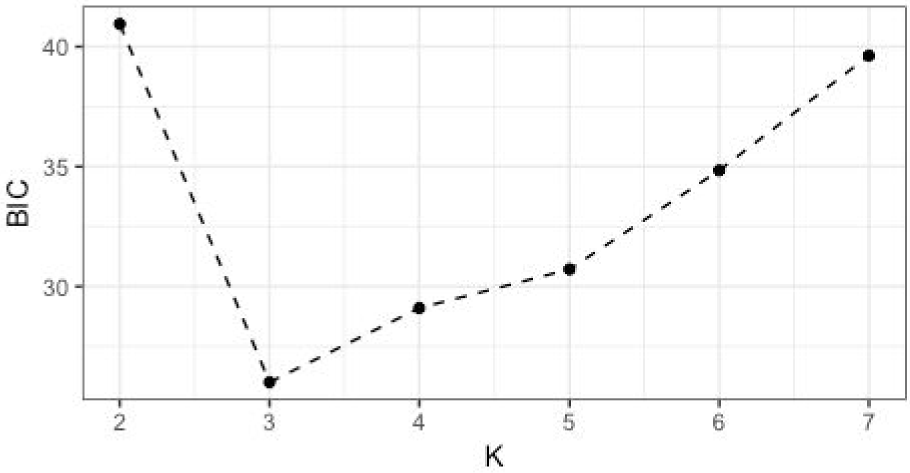

Appendix Figure S10 | BIC for the *K*-Means clustering on Factor 1 in the CLL data. Values of the Bayesian Information Criterion (BIC) for different values of K in the K-means clustering on Factor 1. A minimum is obtained for *K*=3.

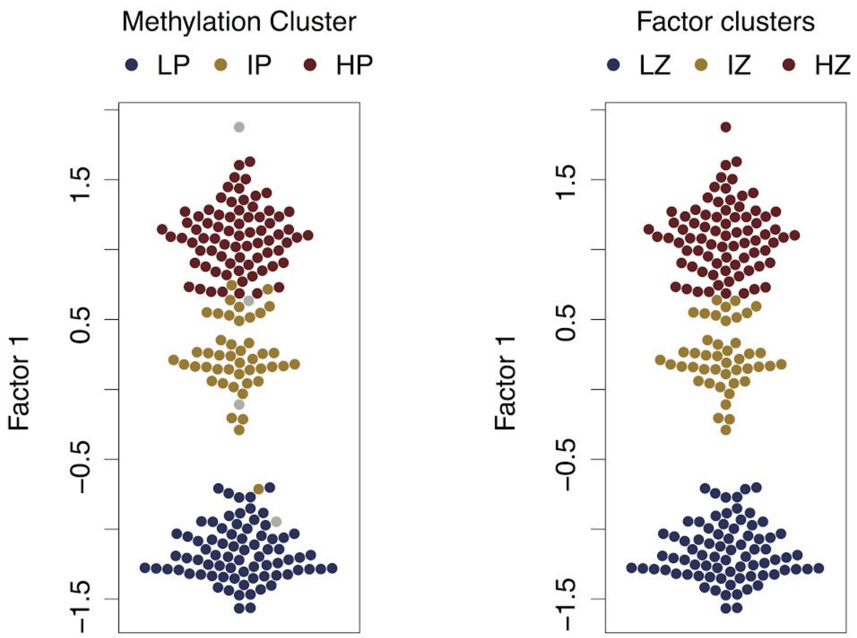

	low Factor 1 (LZ)	intermediate Factor 1 (IZ)	high Factor 1 (HZ)
low-programmed (LP)	76	0	0
intermediate-programmed (IP)	1	42	2
high-programmed (HP)	0	0	75
missing	1	2	1
total	**78**	**44**	**78**
Drug response data present	73 (93.5%)	41 (93.1%)	70 (89.7%)
Methylation data present	77 (98.7%)	43 (97.7%)	76 (97.4%)
Mutation data present	78 (100%)	44 (100%)	78 (100%)
RNAseq data present	54 (69.2 %)	28 (63.6%)	54 (69.2%)

Appendix Figure S11 | Correspondence of patient clusters on Factor 1 with previously described CLL subgroups. Beeswarm plots of Factor 1 colored by previously described CLL subgroups (Oakes et al, 2016) (left) and 3-means clusters (right) as in **Figure 3a**. The table below indicates the sample numbers that fall in each of these clusters as well as the number of samples in each cluster with data available for the given omic type.

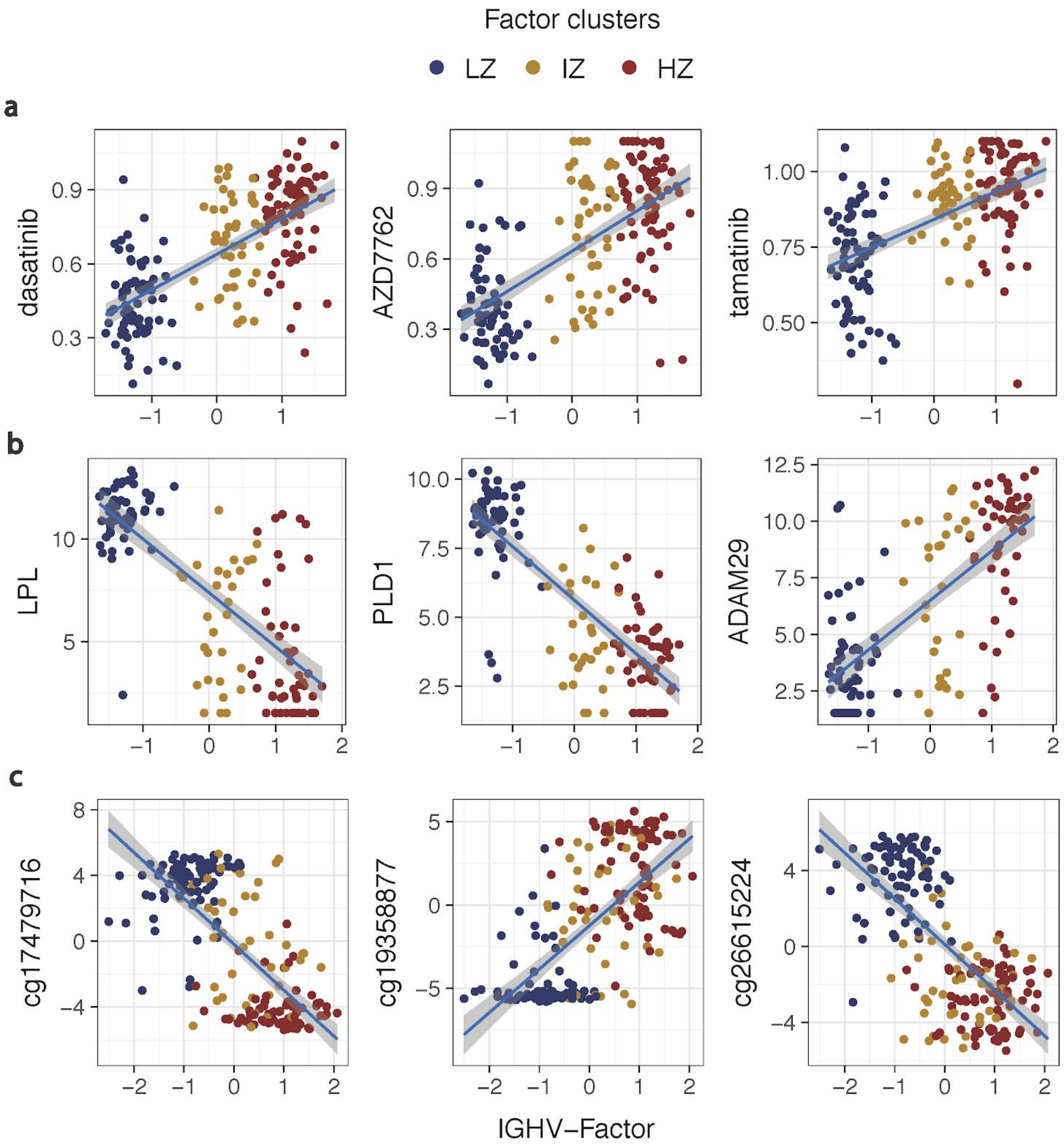

Appendix Figure S12 | Correlation between the continuous IGHV state inferred by MOFA (Factor 1) and individual molecular features. Scatterplots showing the correlation between the continuous Factor 1 inferred by MOFA and molecular features. To avoid circularity, models were re-trained holding out different data modalities in turn: **(a)** drug response, **(b)** gene expression and **(c)** methylation. Colors denote cluster assignments using the factor obtained from the full data set. Displayed are representative features with high absolute loading on the Factor 1 from the full model.

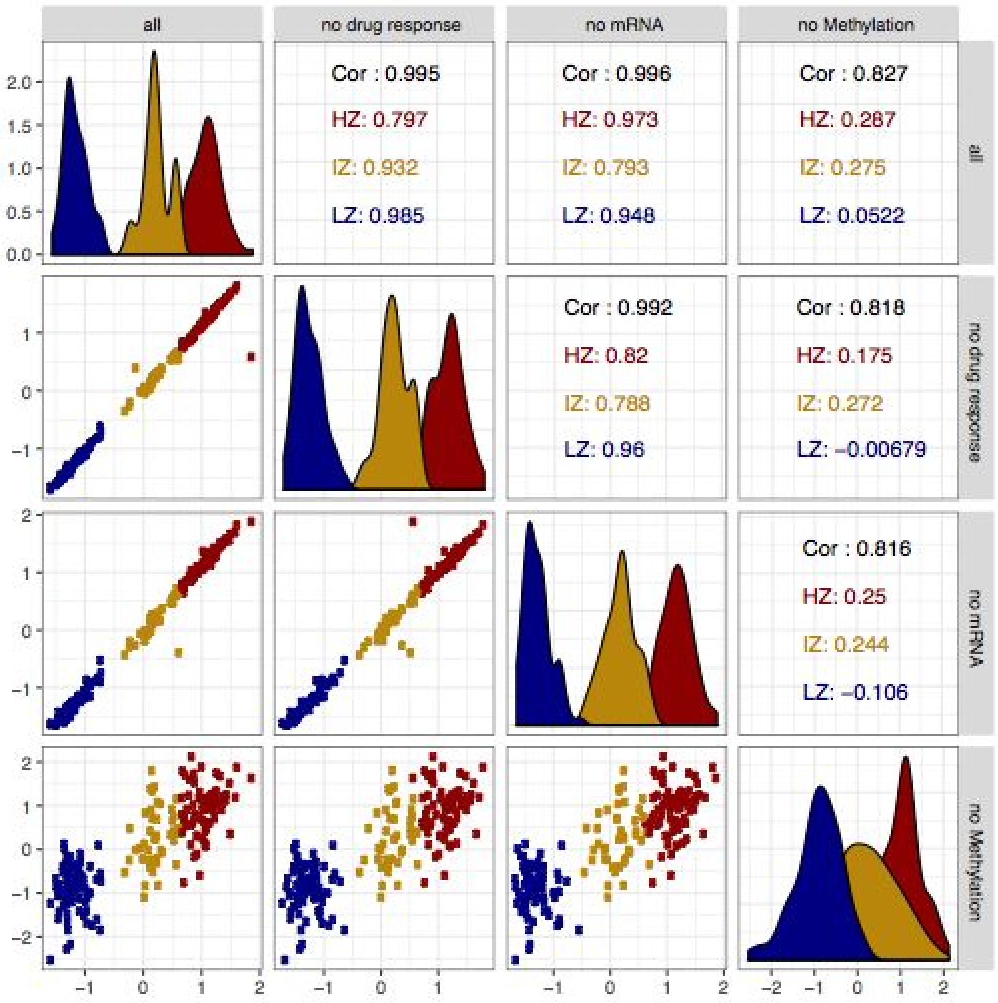

Appendix Figure S13 | Correlation between the continuous IGHV state inferred by MOFA (Factor 1) trained on different subset of data modalities. Scatterplots on the lower panels show the pairwise correlation between the continuous Factor 1 inferred by MOFA when training on all assays, without the drug response assay, mRNA assay and methylation assay, respectively. Colors are based on the clusters on Factor 1 inferred by the full model (trained on all data modalities). The panels on the diagonal show the densities of factor values of the 3 different clusters in each setting and the upper panels denote the overall and within-cluster correlation.

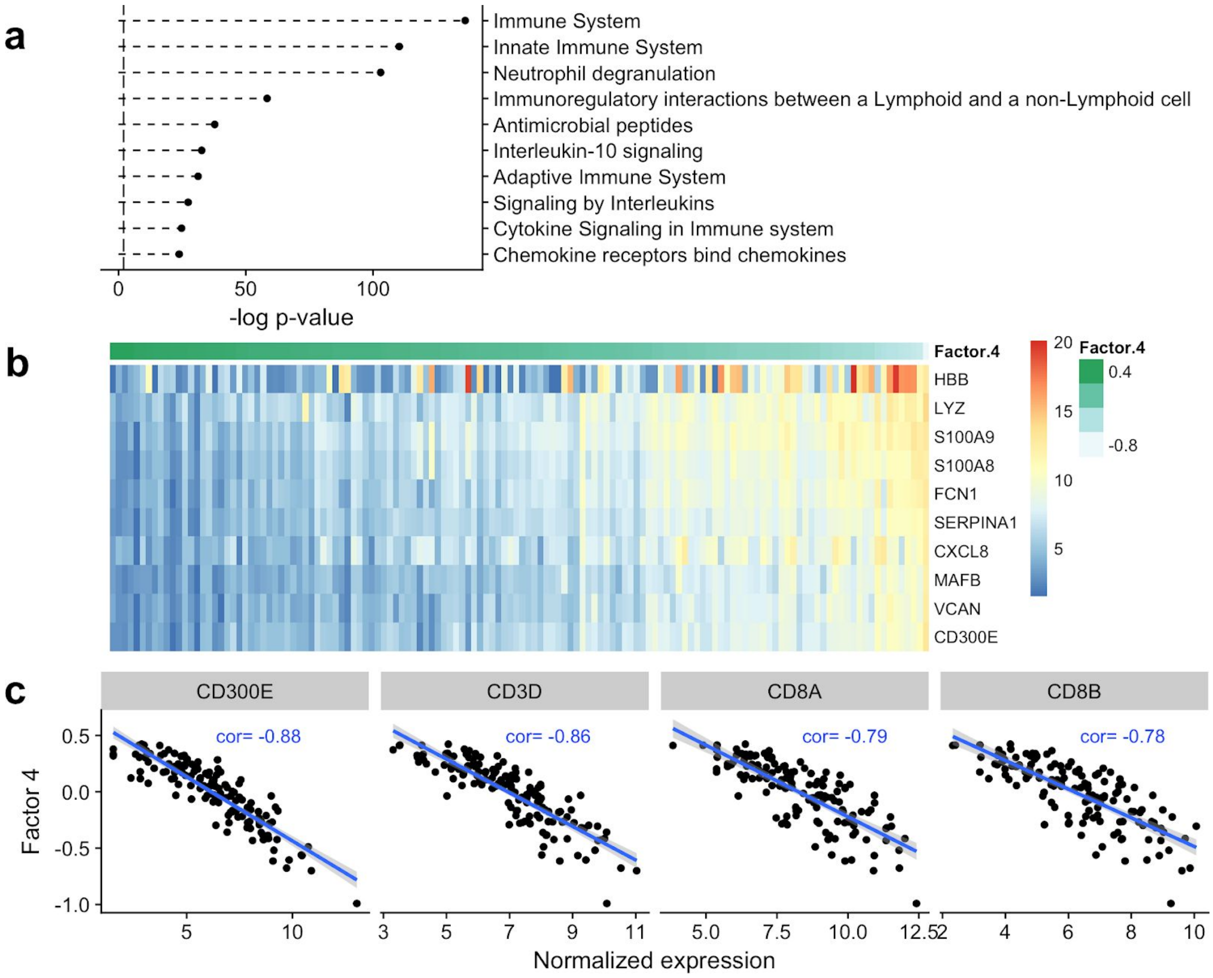

Appendix Figure S14 | Characterization of Factor 4 in the CLL data. (a) Gene sets of the Reactome pathways enriched in the mRNA data (t-test, **Methods,** dashed line represents a FDR of 1%). **(b)** Heatmap of the mRNA data in the top ten features for Factor 4. Samples are ordered along their value on the respective factor as shown on top of the heatmap. **(c)** Scatterplot of the normalized expression of important surface markers of T-cells (CD8A, CD8B, CD3D) and monocytes (CD300E) versus the values on Factor 4.

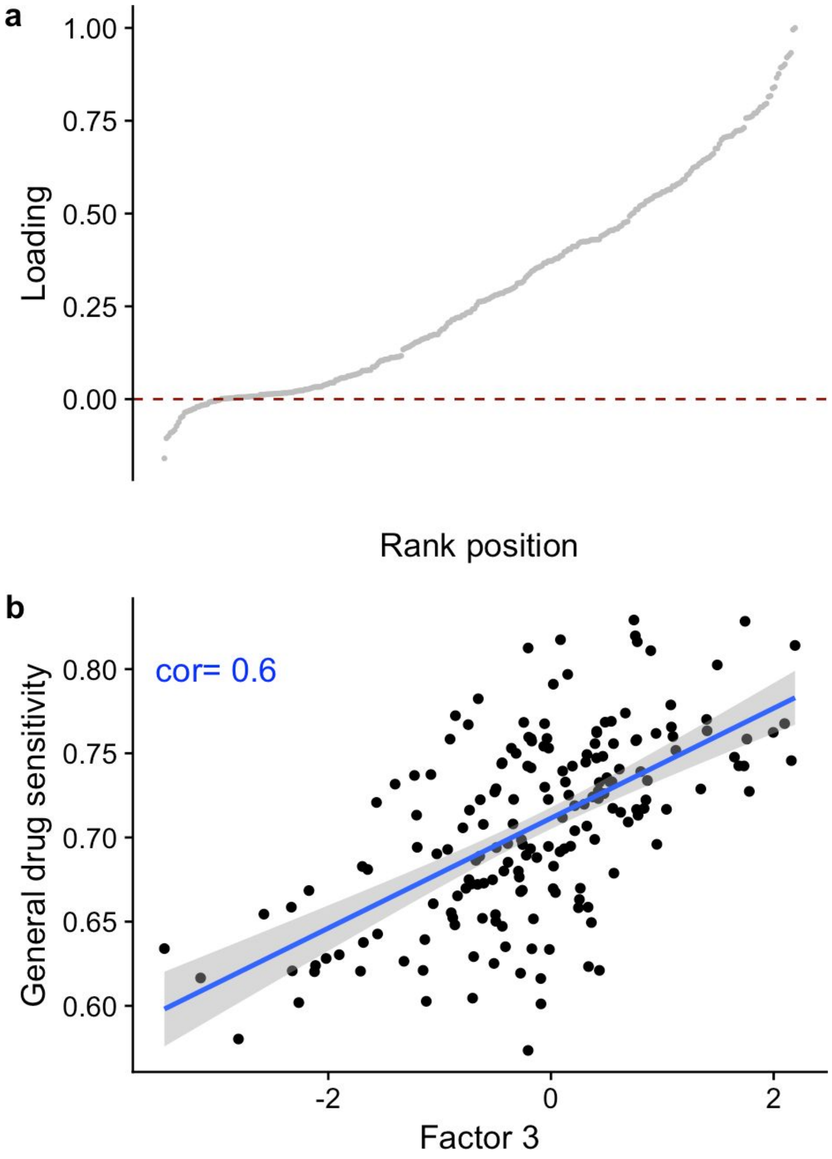

Appendix Figure S15 | Characterization of Factor 3 in the CLL data. (a) Loadings of all drugs and concentrations on Factor 3. **(b)** Scatterplot of Factor 3 versus a general level of drug sensitivity calculated as the mean viability of a sample across all drugs and concentrations.

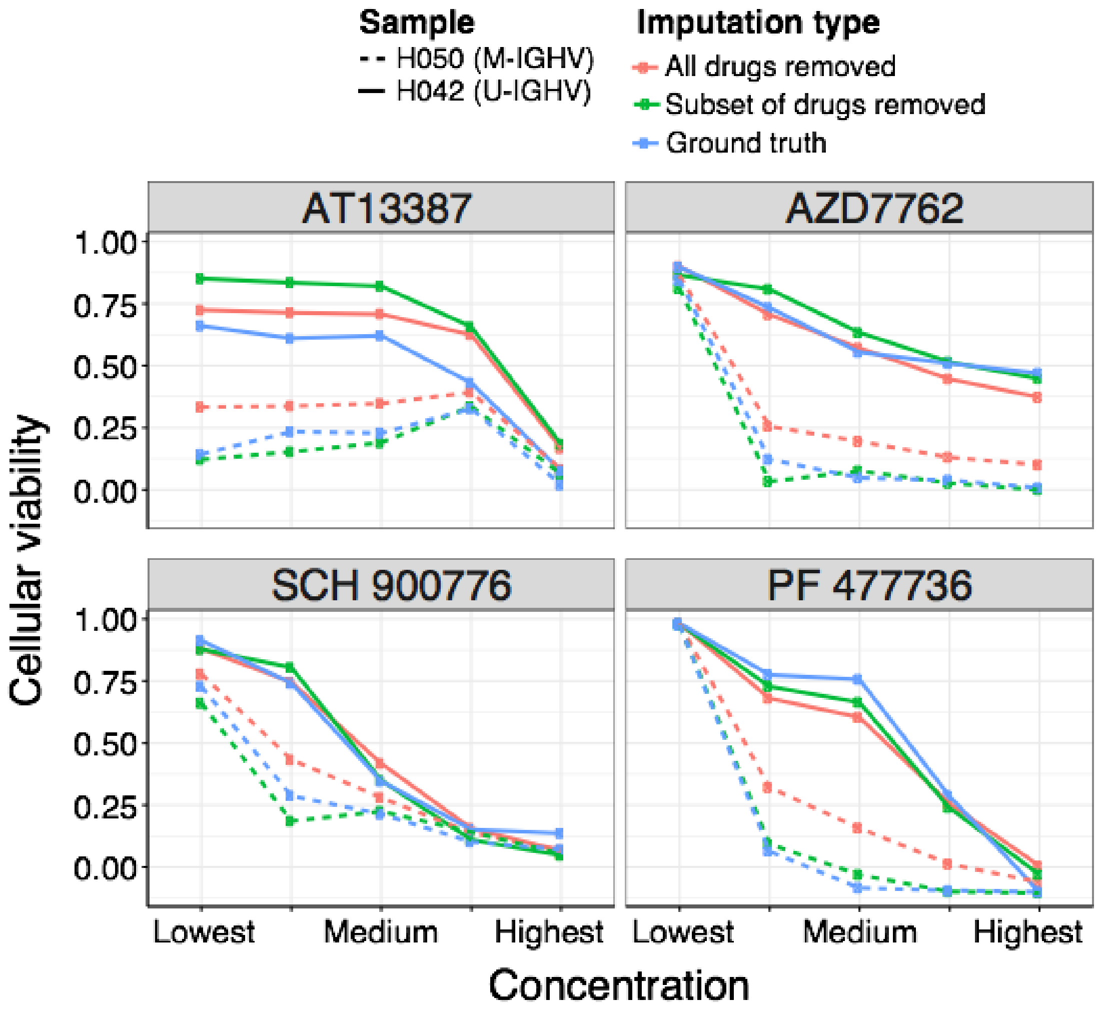

Appendix Figure S16 | Prediction of drug response curves in the CLL data. Prediction of drug response curves for two samples clinically annotated as M-IGHV (H050, dashed line) and U-IGHV (H052, solid line), respectively, for four representative drugs known to be affected by IGHV status. Scatterplots show the predicted drug response curve as cellular viability versus concentration when training a MOFA model removing all drugs from the corresponding patients (red) and removing only the four drugs (green). The true response curve is shown in blue.

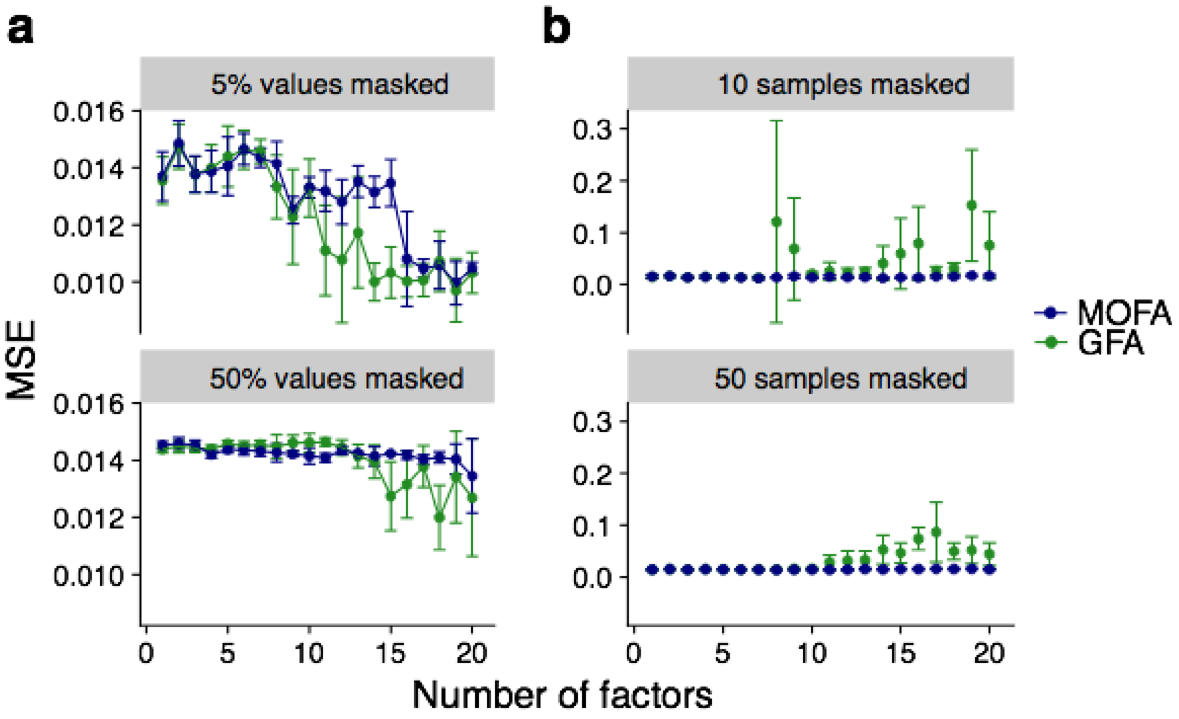

Appendix Figure S17 | Comparison of the accuracy of MOFA and GFA for imputing missing values in the drug response assay of the CLL data. GFA and MOFA models were trained with different numbers of factors. Shown are averages of the mean squared error (MSE) across 5 imputation experiments for different fractions of missing data, considering **(a)** values missing at random (top panel: 5%; bottom pannel: 50%) and **(b)** entire assay missing for samples at random (top panel: N=10; bottom panel: N=50). Error bars denote plus or minus two standard error.

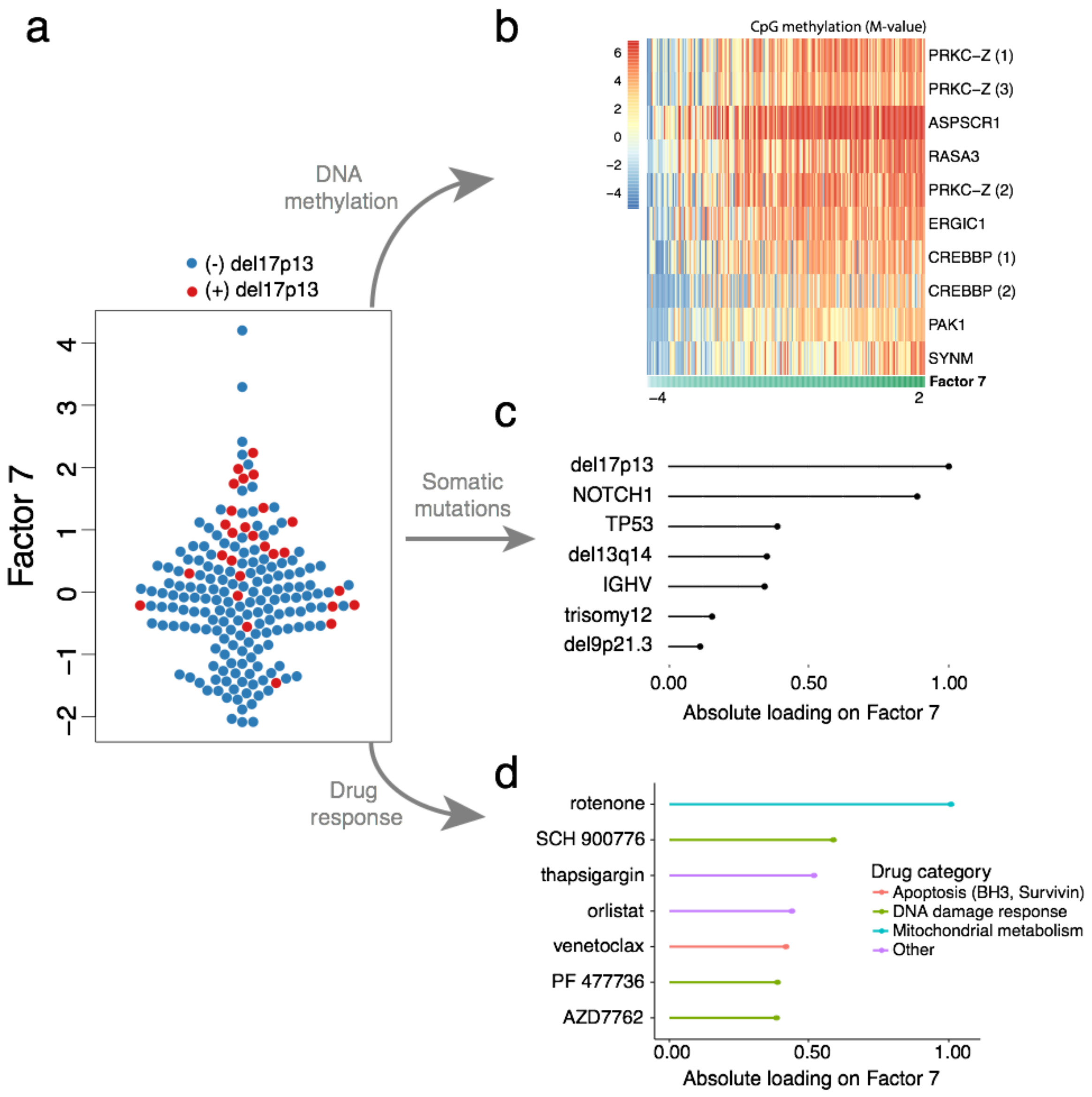

Appendix Figure S18 | Characterisation of Factor 7 in the CLL data. (a) Beeswarm plot with Factor 7 values for each sample. Colors denote the presence or absence of the deletion del17p13. **(b)** Heatmap of methylation (M-value) for CpG sites with the largest loading (matched to overlapping genes). **(c)** Absolute loadings of top features in the somatic mutation data, **(d)** Absolute loadings of the top features in the drug response data.

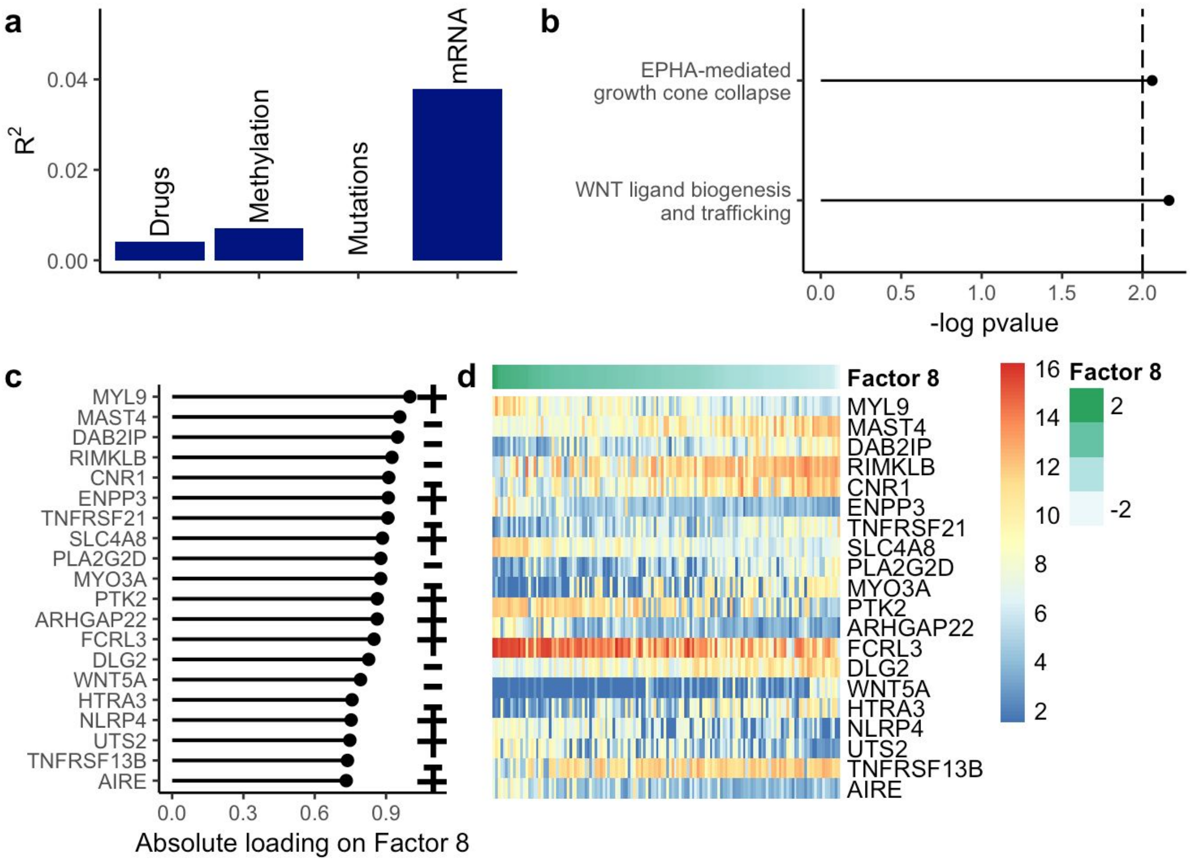

Appendix Figure S19 | Characterization of Factor 8 in the CLL data. (a) Variance explained by Factor 8 in the four assays. **(b)** Gene sets enriched the for the Reactome pathways in the mRNA data at a FDR of 1% (t-test, **Methods**). **(c)** Absolute values for the weights of top 20 genes in the mRNA data, sign indicating the direction of their effect. **(d)** Heatmap of the normalized expression values for the genes shown in **c**, samples are ordered along their values on Factor 8.

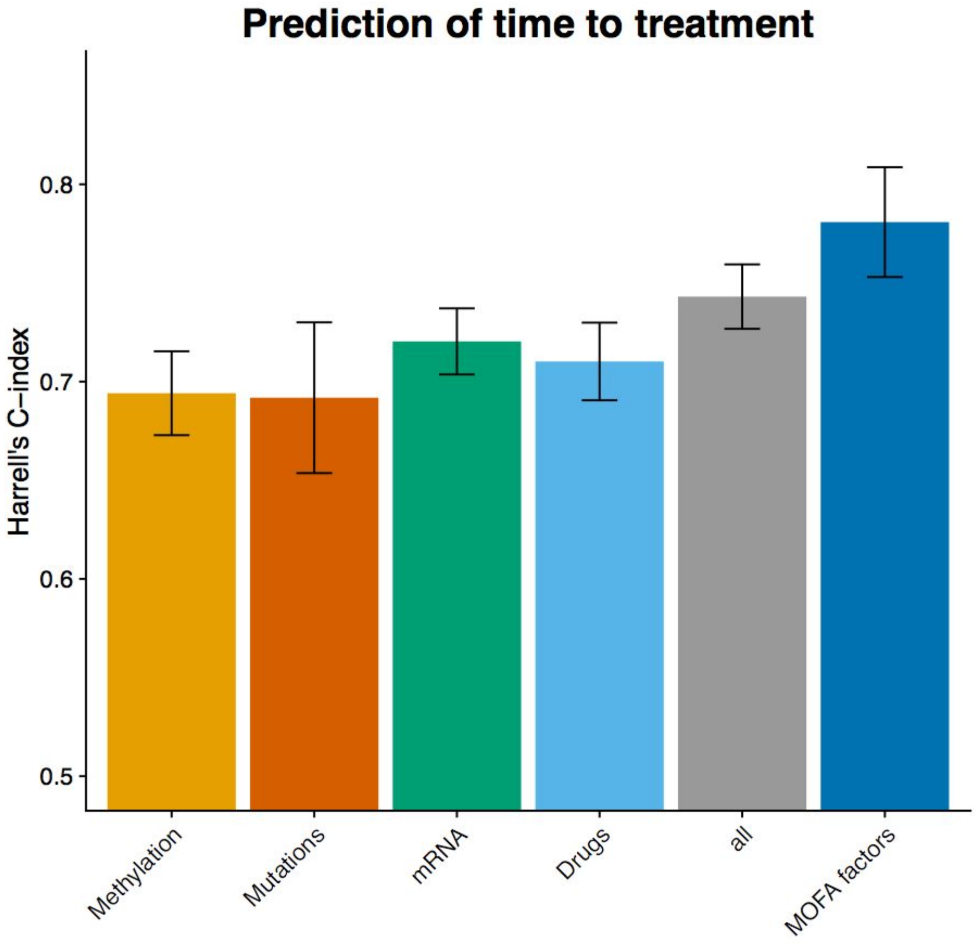

Appendix Figure S20 | Prediction accuracy of time to next treatment using MOFA factors and raw features of the assays in the CLL data. Considered are L_2-penalized Cox models trained on the features of individual assays as well as their superset (*all*). For comparison the result using a Cox model trained on the 10 MOFA factors is shown as in **Figure 4**. The y-axis shows Harrell's C index as a measure of prediction performance. The average value over 5-fold cross-validation is shown with error bars indicating the standard error. Assays with missing values were imputed using the feature-wise mean.

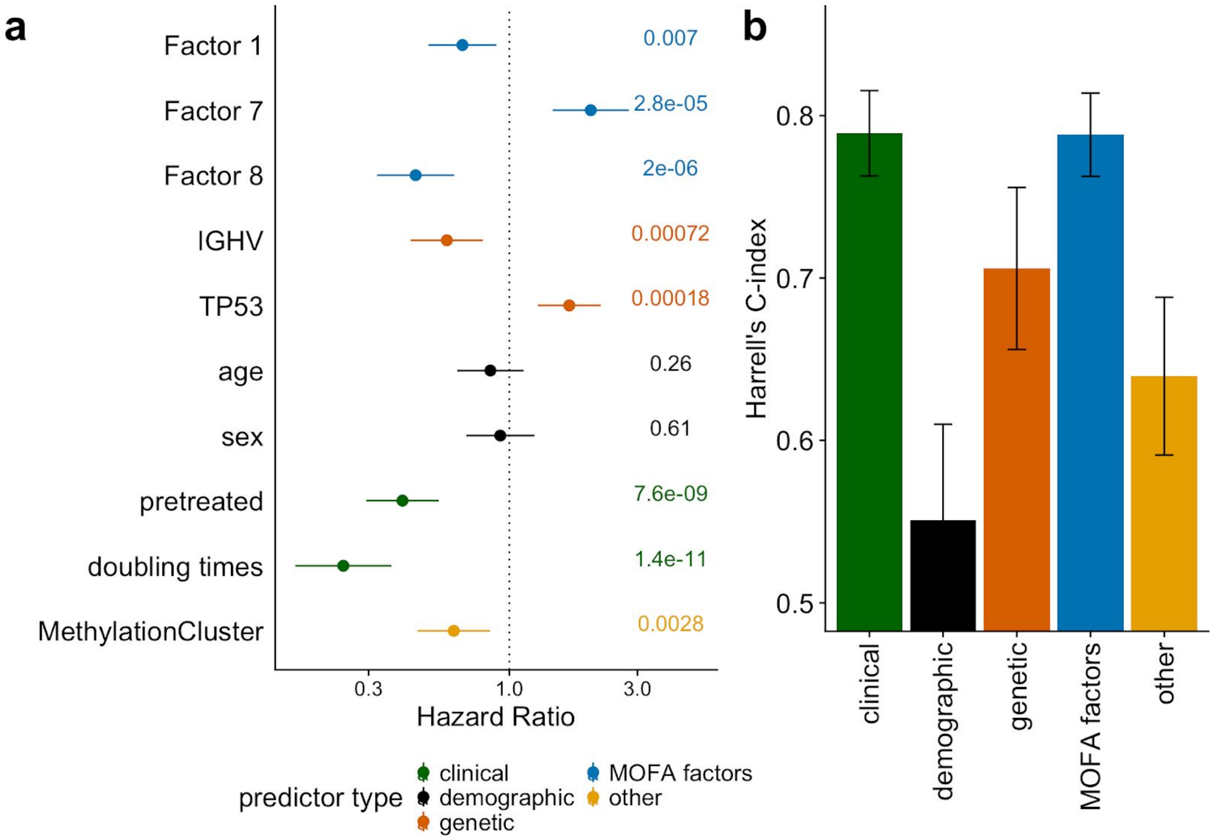

Appendix Figure S21 | Comparison of MOFA factors with clinical covariates in the CLL data. (a) Association of MOFA factors and clinical covariates with time to next treatment using a univariate Cox models for *n*=76 samples, for which the clinical information was available. Error bars denote 95% confidence intervals. Numbers on the right denote p-values for each predictor. **(b)** Prediction accuracy of time to treatment using multivariate Cox regression trained using the 10 factors derived using MOFA as well as the selected clinical predictors in panel **a**. Shown are average values of Harrell's C index from 5-fold cross-validation. Error bars denote standard error of the mean.

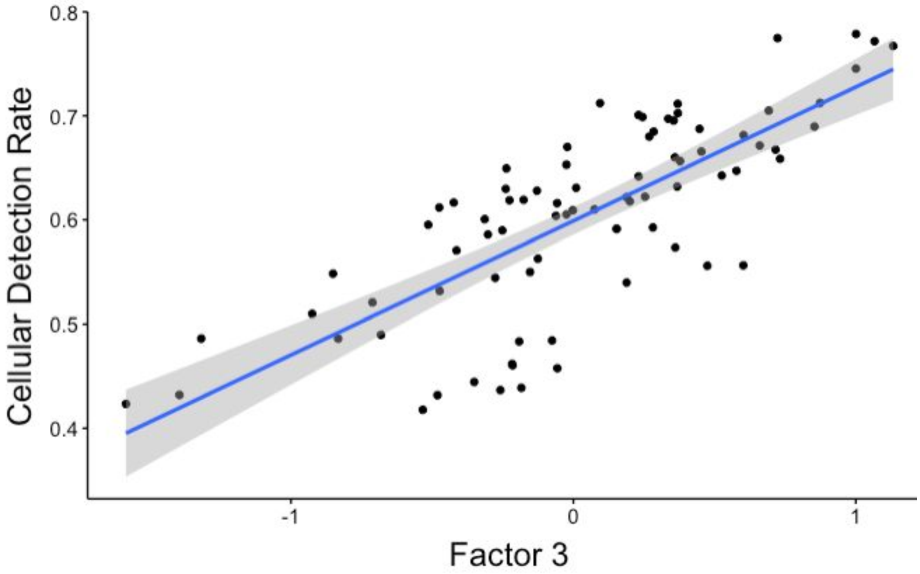

Appendix Figure S22 | Characterisation of Factor 3 in the scMT data. Scatterplot depicting the correlation between Factor 3 and the Cellular Detection Rate, a known technical factor in single-cell RNA-seq data that corresponds to the fraction of expressed genes.

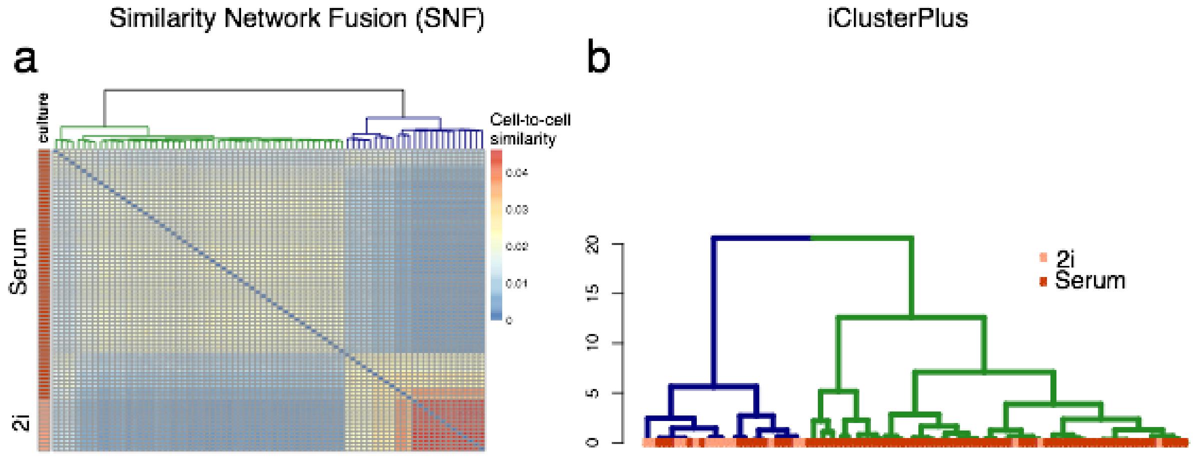

Appendix Figure S23 | Multi-omics clustering applied to scMT data set. (a) Similarity matrix and dendogram obtained using Similarity Network Fusion (Wang et al. 2014). **(b)** Dendrogram obtained using *iClusterPlus* (Mo et al. 2013) with two clusters (*K*=1, model selection by optimal BIC). The cells at the leaves are colored by culture condition.

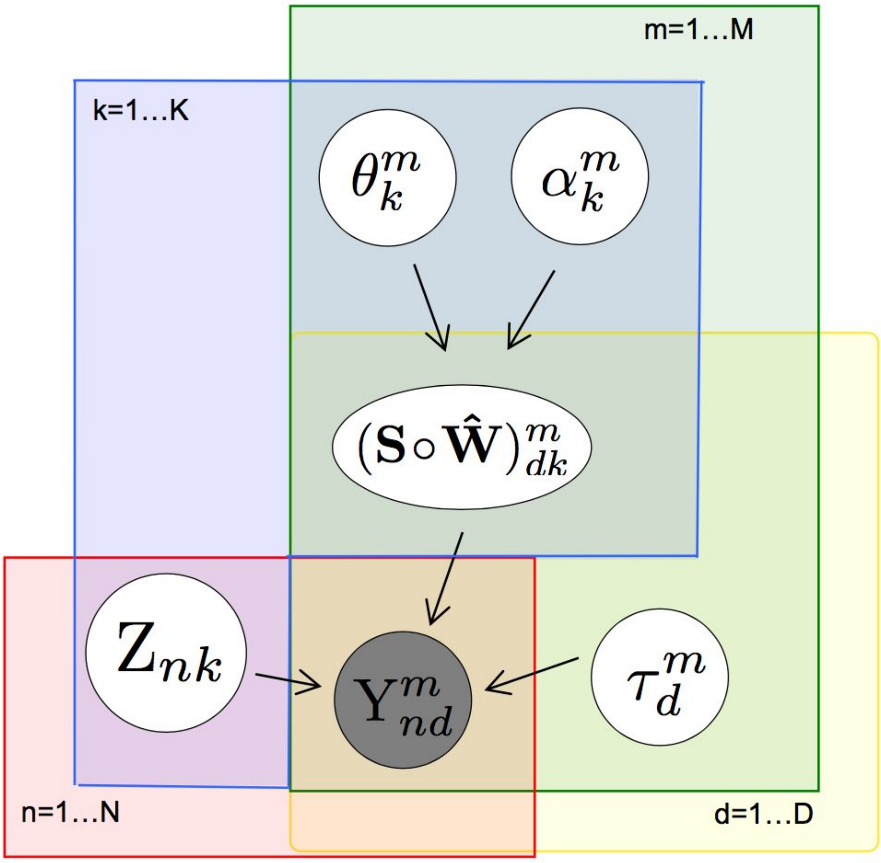

Appendix Figure S24 | Graphical model representation of MOFA. Grey-filled nodes denote observed variables whereas white-filled nodes denote unobserved variables that are inferred by the model. Y denotes the observed data matrices, Z denotes latent factors and $S \circ \widehat{W}$ denotes the model weights with a spike-and-slab prior, implemented as the product of a Bernoulli variable S and a Gaussian variable $\widehat{W}$. θ represents the sparsity parameter of the spike-and-slab prior and α corresponds to the view- and factor-wise Automatic Relevance Determination prior. τ represents the precision of the normally-distributed noise. N is the number of samples, M is the number of views, D is the number of features in the m-th view and K is the number of latent factors.

7 Supplementary Tables

1. Simulation settings to validate the ability to learn the number of active factors (Appendix Figure S1)

likelihood	# factors	# features	# views	# samples	Missingness (%)
gaussian	(5,10,...,60)	5000	3	100	0
gaussian	10	(100,500,...,10000)	3	100	0
gaussian	10	5000	(1,3,...,21)	100	0
gaussian	10	5000	3	100	(0,5,10,...,90)

2. Simulation settings to validate non-gaussian likelihoods (Appendix Figure S2-3)

likelihood	# factors	# features	# views	# samples	Missingness (%)
bernoulli	10	5000	3	100	0
poisson	10	5000	3	100	0

3. Simulations settings for the GFA and iCluster comparison (Appendix Figure S4-5)

Likelihoods	# factors	# features	# views	# samples	Missingness (%)
gaussian, bernoulli and poisson	10	5000	3	100	5

Appendix Table S1 | Parameters for simulation settings

**Immune Response**
Interleukin-6 signaling
Interleukin-7 signaling
Cytokine Signaling in Immune system
Adaptive Immune System
Innate Immune System
Immune System
Immunoregulatory interactions between a Lymphoid and a non-Lymphoid cell
TCR signaling
Downstream TCR signaling
Phosphorylation of CD3 and TCR zeta chains
Translocation of ZAP-70 to Immunological synapse
Interleukin-1 signaling
Signaling by Interleukins
Interleukin-2 signaling
Interleukin-3, 5 and GM-CSF signaling
Diseases of Immune System
Interleukin-6 family signaling
Interleukin-10 signaling
Interleukin-4 and 13 signaling
IL-6-type cytokine receptor ligand interactions
Interleukin receptor SHC signaling
**Cellular Stress/Senescence**
Telomere Maintenance
Packaging Of Telomere Ends
Polymerase switching on the C-strand of the telomere
Processive synthesis on the C-strand of the telomere
Telomere C-strand (Lagging Strand) Synthesis
Activation of ATR in response to replication stress
Extension of Telomeres
Cellular responses to stress
Oxidative Stress Induced Senescence
Senescence-Associated Secretory Phenotype (SASP)
Cellular Senescence
Formation of Senescence-Associated Heterochromatin Foci (SAHF)
Oncogene Induced Senescence
DNA Damage/Telomere Stress Induced Senescence
Regulation of HSF1-mediated heat shock response
HSF1 activation
Cellular response to heat stress
Attenuation phase
HSF1-dependent transactivation

**RNA regulation**
RNA Polymerase II HIV Promoter Escape
SIRT1 negatively regulates rRNA Expression
ERCC6 (CSB) and EHMT2 (G9a) positively regulate rRNA expression
NoRC negatively regulates rRNA expression
Regulation of mRNA stability by proteins that bind AU-rich elements
RNA Polymerase I, RNA Polymerase III, and Mitochondrial Transcription
Positive epigenetic regulation of rRNA expression
B-WICH complex positively regulates rRNA expression
Negative epigenetic regulation of rRNA expression
Transcriptional regulation by small RNAs
RNA Polymerase II Pre-transcription Events
RNA Polymerase I Promoter Opening
RNA Polymerase I Transcription Initiation
RNA Polymerase I Promoter Escape
RNA Polymerase II Promoter Escape
RNA Polymerase I Chain Elongation
RNA Polymerase II Transcription Pre-Initiation And Promoter Opening
RNA Polymerase III Chain Elongation
RNA Polymerase I Promoter Clearance
RNA Polymerase II Transcription Termination
RNA Polymerase II Transcription
RNA Polymerase I Transcription Termination
RNA Polymerase I Transcription
RNA Polymerase III Transcription Termination
RNA Polymerase III Transcription
RNA Polymerase III Abortive And Retractive Initiation
RNA Polymerase II Transcription Initiation
RNA Polymerase II Transcription Elongation
RNA Polymerase II Transcription Initiation And Promoter Clearance
RNA Polymerase III Transcription Initiation
RNA Polymerase III Transcription Initiation From Type 1 Promoter
RNA Polymerase III Transcription Initiation From Type 2 Promoter
RNA Polymerase III Transcription Initiation From Type 3 Promoter

Appendix Table S2 | Coarse-grain categories of gene set used in Figure 2

Publication	Inference	Group-wise sparsity	Feature-wise sparsity	Missing values	Likelihood	Noise model
Shen et al, 2009	EM, grid search	different L_1- penalties	L_1-penalty	No	Gaussian	Hetero-scedastic
Mo et al, 2013	EM, grid search	different L_1- penalties	L_1-penalty	No	Gaussian, Poisson, Bernoulli Multinomial	Hetero-scedastic
Virtanen et al, 2012	VB	ARD	None	No	Gaussian	Homo-scedastic
Klami et al, 2014	VB	ARD	None	No	Gaussian	Homo-scedastic
Bunte et al, 2016	Gibbs	ARD	Spike and Slab	No	Gaussian	Homo-scedastic
Hore et al, 2016	VB	None	Spike and Slab	Yes	Gaussian	Hetero-scedastic
Remes et al, 2016	VB	ARD	None	No	Gaussian	Homo-scedastic
Zhao et al, 2015	Gibbs	ARD	Three-parameter beta prior	No	Gaussian	Hetero-scedastic
Leppäaho et al., 2017	Gibbs	ARD	Spike and Slab	Yes	Gaussian	Homo-scedastic
MOFA	VB	ARD	Spike and Slab	Yes	Gaussian, Poisson, Bernoulli	Hetero-scedastic

Appendix Table S3 | Overview of GFA and iCluster methods

Abbreviations used: VB (variational Bayes inference), Gibbs (Gibbs sampling based inference), ARD (Automatic Relevance Determination), EM (Expectation-Maximization)
