# Supplementary material for: Multi‐Omics Factor Analysis—a framework for unsupervised integration of multi‐omics data sets
Source: Mol Syst Biol. 2018 Jun 20;14(6):e8124. doi: 10.15252/msb.20178124 (PMC6010767; doi:10.15252/msb.20178124)
Supplement: Supplementary file 2 — Expanded View Figures PDF [file MSB-14-e8124-s002.pdf]

Expanded View Figures

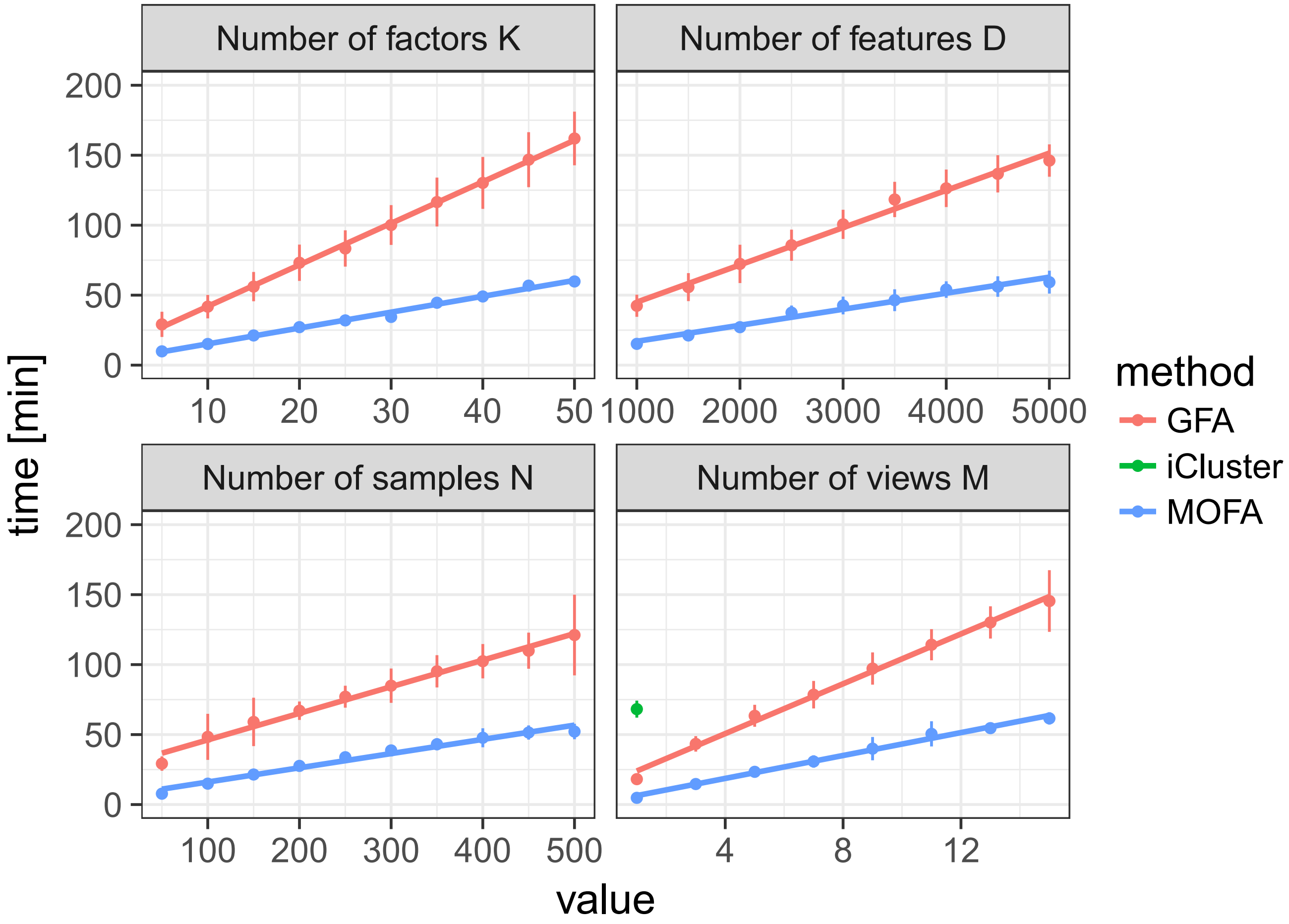

Figure EV1. Scalability of MOFA, GFA and iCluster.

Time required for model training for GFA (red), MOFA (blue) and iCluster (green) as a function of number of factors K, number of features D, number of samples N and number of views M. Baseline parameters were $M = 3$, $K = 10$, $D = 1{,}000$ and $N = 100$ and 5% missing values. Shown are average time across 10 trials, and error bars denote standard deviation. iCluster is only shown for the lowest M as all other settings require on average more than 200 min for training.

© 2018 The Authors

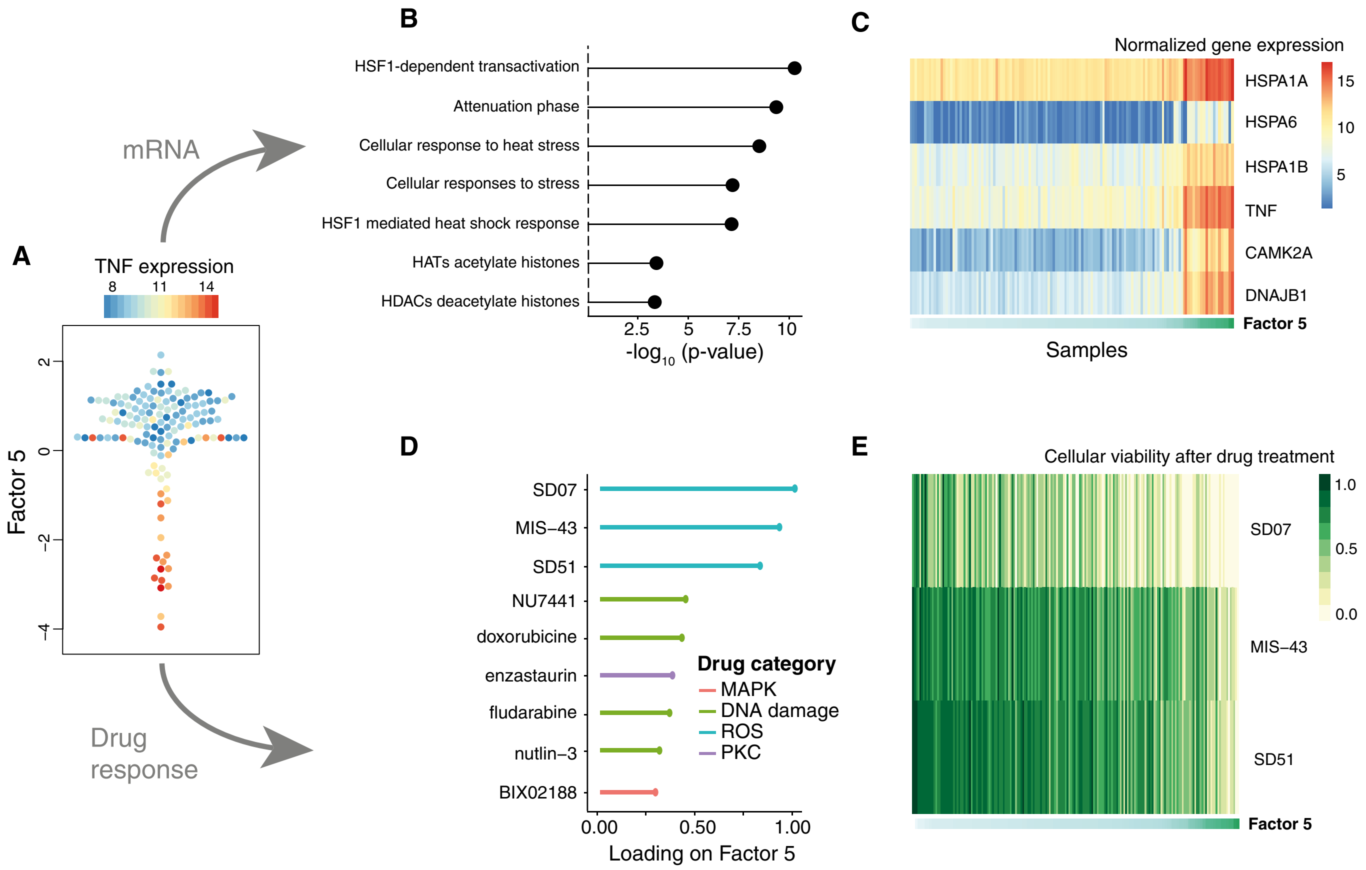

Figure EV2. Characterization of Factor 5 (oxidative stress response factor) in the CLL data.

A Beeswarm plot of Factor 5. Colours denote the expression of TNF, an inflammatory stress marker.
B Gene set enrichment analysis for the top Reactome pathways in the mRNA data (*t*-test, Materials and Methods).
C Heatmap of gene expression values for the six genes with largest loading. Samples are ordered by their factor values.
D Scaled loadings for the top drugs with the largest loading, annotated by target category.
E Heatmap of drug response values for the top three drugs with largest loading.

Figure EV3. Prediction of IGHV status based on Factor 1 in the CLL data and validation on outlier cases on independent assays.

A Beeswarm plot of Factor 1 with colours denoting agreement between predicted and clinical labels as in (B).
B Pie chart showing total numbers for agreement of imputed labels with clinical label.
C Sample-to-sample correlation matrix based on drug response data.
D Sample-to-sample correlation matrix based on methylation data.
E Drug response to ONO-4509 (not included in the training data): Boxplots for the viability values in response to ONO-4509. The three outlier samples are shown in the middle; on the left and right, the viabilities of the other M-CLL and U-CLL samples are shown, respectively. The panels show different drug concentrations tested. Boxes represent the first and third quartiles of the values for M-CLL and U-CLL samples, for individual patients the single value.
F Whole exome sequencing data on IGHV genes (not included in the training data): the number of mutations found on IGHV genes using whole exome sequencing is shown on the *y*-axis, separately for U-CLL and M-CLL samples. The three outlier samples are labelled.

▶

© 2018 The Authors

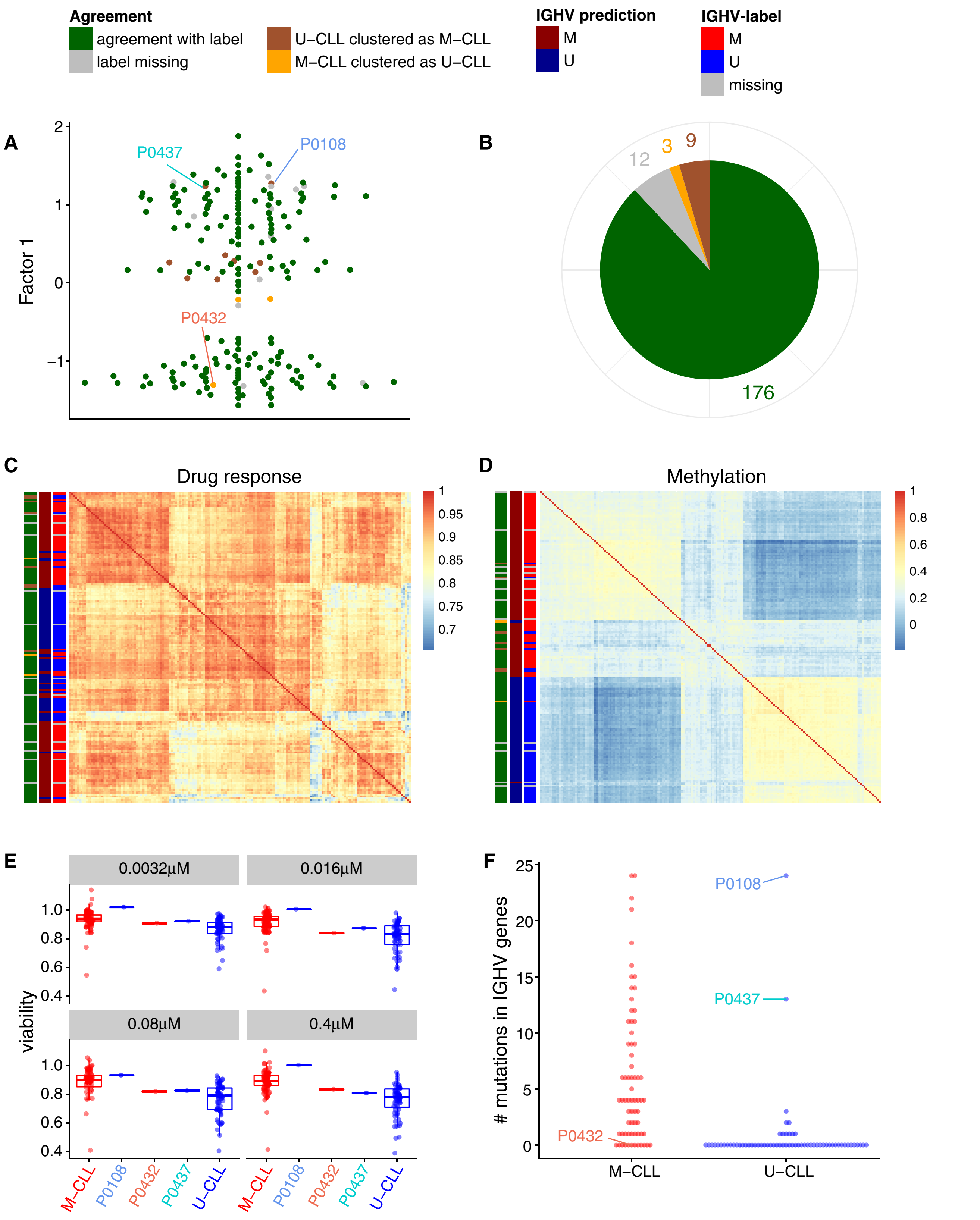

Figure EV3.

 © 2018 The Authors

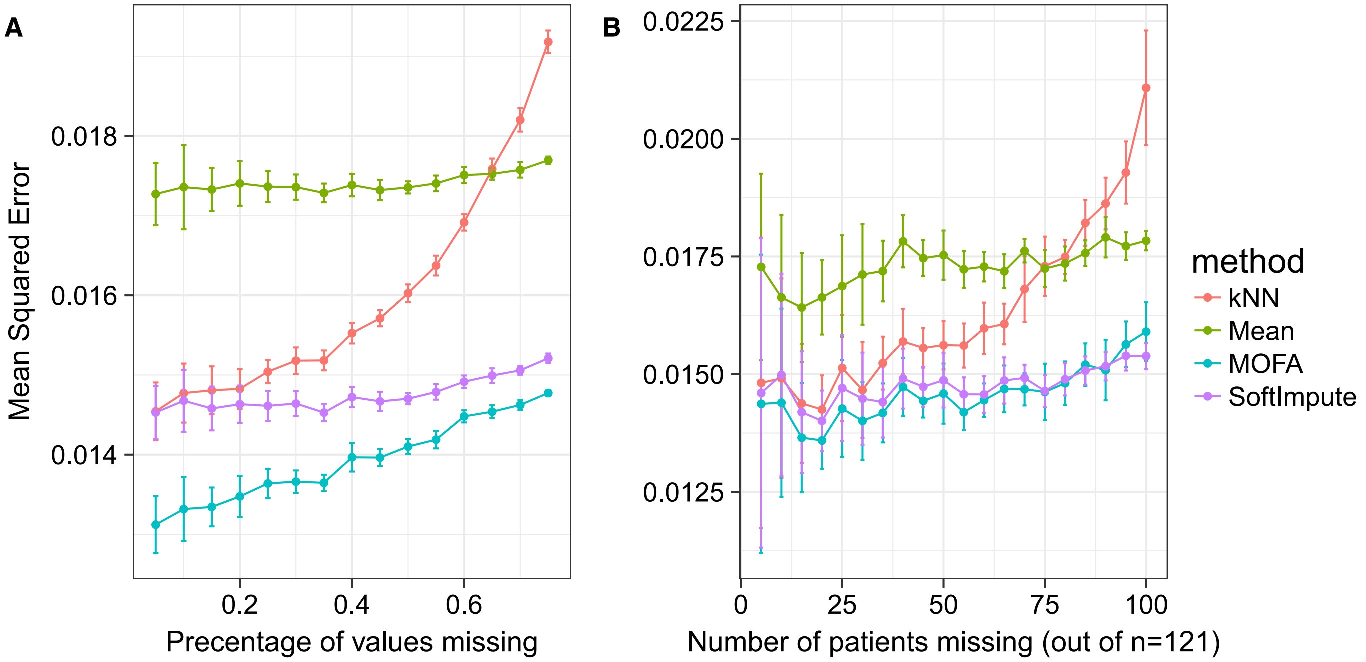

Figure EV4. Imputation of missing values in the drug response assay of the CLL data.

A, B Considered were MOFA, SoftImpute, imputation by feature-wise mean (Mean) and k-nearest neighbour (kNN). Shown are averages of the mean squared error (MSE) across 15 imputation experiments for increasing fractions of missing data, considering (A) values missing at random and (B) entire assay missing for samples at random. Error bars denote plus or minus two standard error.

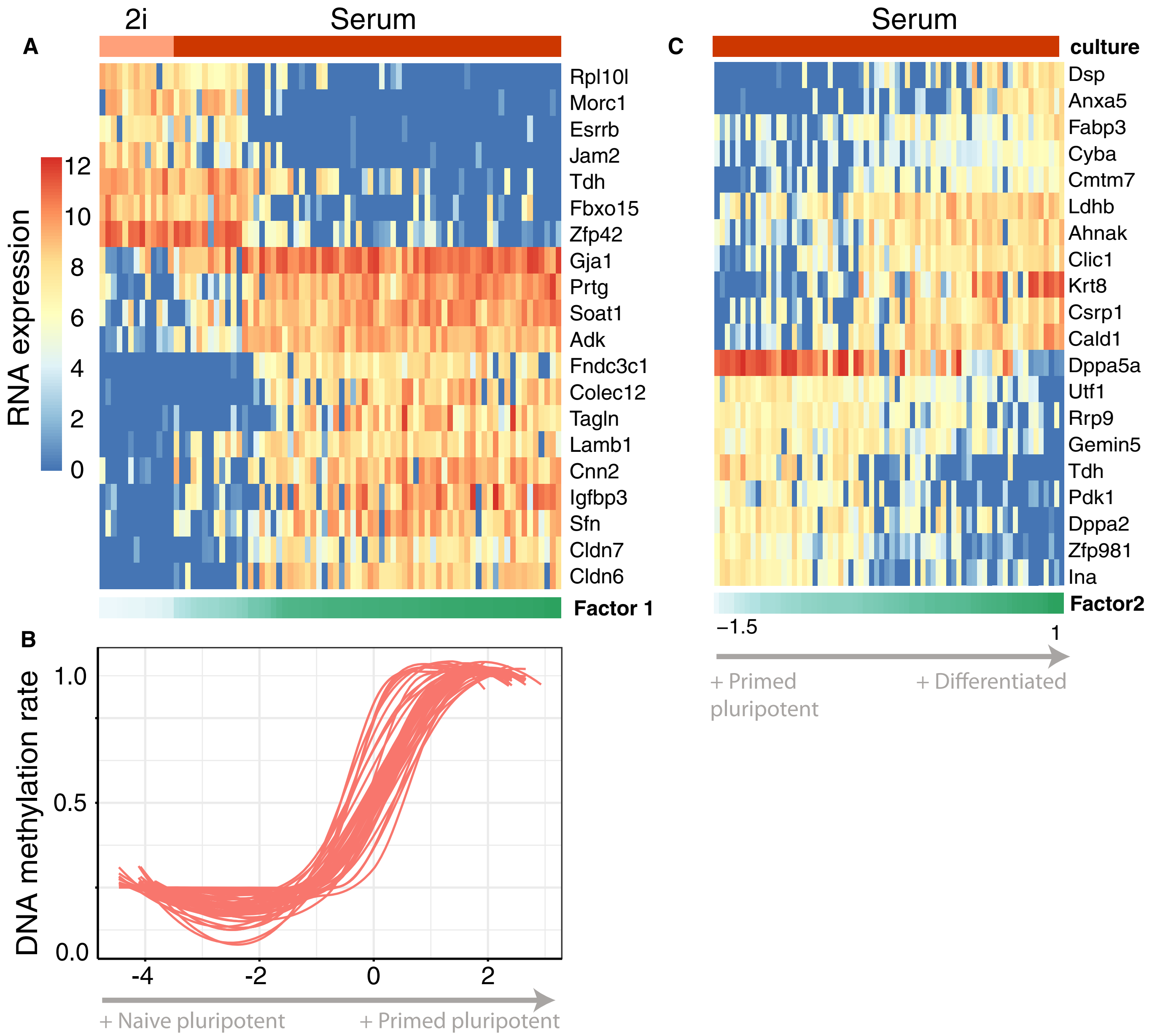

Figure EV5. Transcriptomic and epigenetic changes associated with Factor 1 in the scMT data.

A RNA expression changes for the top 20 genes with largest weight on Factor 1.

B DNA methylation rate changes for the top 20 CpG sites with largest weight. Shown is a non-linear loess regression model fit per CpG site.

C RNA expression changes for the top 20 genes with largest weight on Factor 2.

© 2018 The Authors
